# Analysis and Optimization of Equitable US Cancer Clinical Trial Center Access by Travel Time

**DOI:** 10.1001/jamaoncol.2023.7314

**Published:** 2024-03-21

**Authors:** Hassal Lee, Alexander Shakeel Bates, Shawneequa Callier, Michael Chan, Nyasha Chambwe, Andrea Marshall, Mary Beth Terry, Karen Winkfield, Tobias Janowitz

**Affiliations:** 1 Cold Spring Harbor Laboratory, Cold Spring Harbor, New York; 2 Department of Neurobiology and Howard Hughes Medical Institute, Harvard Medical School, Boston, Massachusetts; 3 Department of Clinical Research and Leadership, School of Medicine and Health Sciences, The George Washington University, Washington, DC; 4 Center for Research on Genomics and Global Health, National Human Genome Research Institute, National Institutes of Health, Bethesda, Maryland; 5 Institute of Molecular Medicine, Feinstein Institutes for Medical Research, Manhasset, New York; 6 Warwick Clinical Trials Unit, University of Warwick, Coventry, United Kingdom; 7 Mailman School of Public Health, Columbia University, New York, New York; 8 Herbert Irving Comprehensive Cancer Center, Columbia University Irving Medical Center, New York, New York; 9 Meharry-Vanderbilt Alliance, Vanderbilt University Medical Center, Nashville, Tennessee; 10 Northwell Health Cancer Institute, Manhasset, New York

## Abstract

**Importance:**

Racially minoritized and socioeconomically disadvantaged populations are currently underrepresented in clinical trials. Data-driven, quantitative analyses and strategies are required to help address this inequity.

**Objective:**

To systematically analyze the geographical distribution of self-identified racial and socioeconomic demographics within commuting distance to cancer clinical trial centers and other hospitals in the US.

**Design, Setting, and Participants:**

This longitudinal quantitative study used data from the US Census 2020 Decennial and American community survey (which collects data from all US residents), OpenStreetMap, National Cancer Institute–designated Cancer Centers list, Nature Index of Cancer Research Health Institutions, National Trial registry, and National Homeland Infrastructure Foundation-Level Data. Statistical analyses were performed on data collected between 2006 and 2020.

**Main Outcomes and Measures:**

Population distributions of socioeconomic deprivation indices and self-identified race within 30-, 60-, and 120-minute 1-way driving commute times from US cancer trial sites. Map overlay of high deprivation index and high diversity areas with existing hospitals, existing major cancer trial centers, and commuting distance to the closest cancer trial center.

**Results:**

The 78 major US cancer trial centers that are involved in 94% of all US cancer trials and included in this study were found to be located in areas with socioeconomically more affluent populations with higher proportions of self-identified White individuals (+10.1% unpaired mean difference; 95% CI, +6.8% to +13.7%) compared with the national average. The top 10th percentile of all US hospitals has catchment populations with a range of absolute sum difference from 2.4% to 35% from one-third each of Asian/multiracial/other (Asian alone, American Indian or Alaska Native alone, Native Hawaiian or Other Pacific Islander alone, some other race alone, population of 2 or more races), Black or African American, and White populations. Currently available data are sufficient to identify diverse census tracks within preset commuting times (30, 60, or 120 minutes) from all hospitals in the US (N = 7623). Maps are presented for each US city above 500 000 inhabitants, which display all prospective hospitals and major cancer trial sites within commutable distance to racially diverse and socioeconomically disadvantaged populations.

**Conclusion and Relevance:**

This study identified biases in the sociodemographics of populations living within commuting distance to US-based cancer trial sites and enables the determination of more equitably commutable prospective satellite hospital sites that could be mobilized for enhanced racial and socioeconomic representation in clinical trials. The maps generated in this work may inform the design of future clinical trials or investigations in enrollment and retention strategies for clinical trials; however, other recruitment barriers still need to be addressed to ensure racial and socioeconomic demographics within the geographical vicinity of a clinical site can translate to equitable trial participant representation.

## Key Points

**Question:**

What are the characteristics of populations close to high-volume cancer clinical trial sites and other hospitals in the US?

**Findings:**

This study indicates that the most active US cancer trial sites (n = 78) exist close to socioeconomically more affluent populations with higher proportions of self-identified White individuals than the nationwide average. Modeling of population data identified hospitals within commutable distance to Asian/multiracial/other, Black or African American, White, and socioeconomically disadvantaged populations in cities across the US.

**Meaning:**

These results suggest racial and socioeconomic disparities in commuting distance to US cancer clinical trial sites and identified prospective satellite trial sites that are located close to diverse populations.

## Teaser

This study investigates the geographical distribution of self-identified racial and socioeconomic demographics within commuting distance to cancer clinical trial centers and other hospitals.

## Introduction

Clinical research must improve care for everybody.^1,2^ Minoritized and socioeconomically disadvantaged populations are underrepresented in clinical trials.^3^ This may reduce the generalizability of trial results and propagate health disparities.^4^ Contributors to inequitable trial participation include individual-level factors and structural factors.^5^

Socioeconomic deprivation and travel time to trial centers can impair trial participation. Data on these parameters and population data on self-identified race exist, but their interrelation with clinical research facilities has not been systematically analyzed. Here, we investigate the demographics of the catchment areas of high-volume US trial sites and map potential trial enrollment sites located in diverse population areas.

## Methods

**Figure 1.**
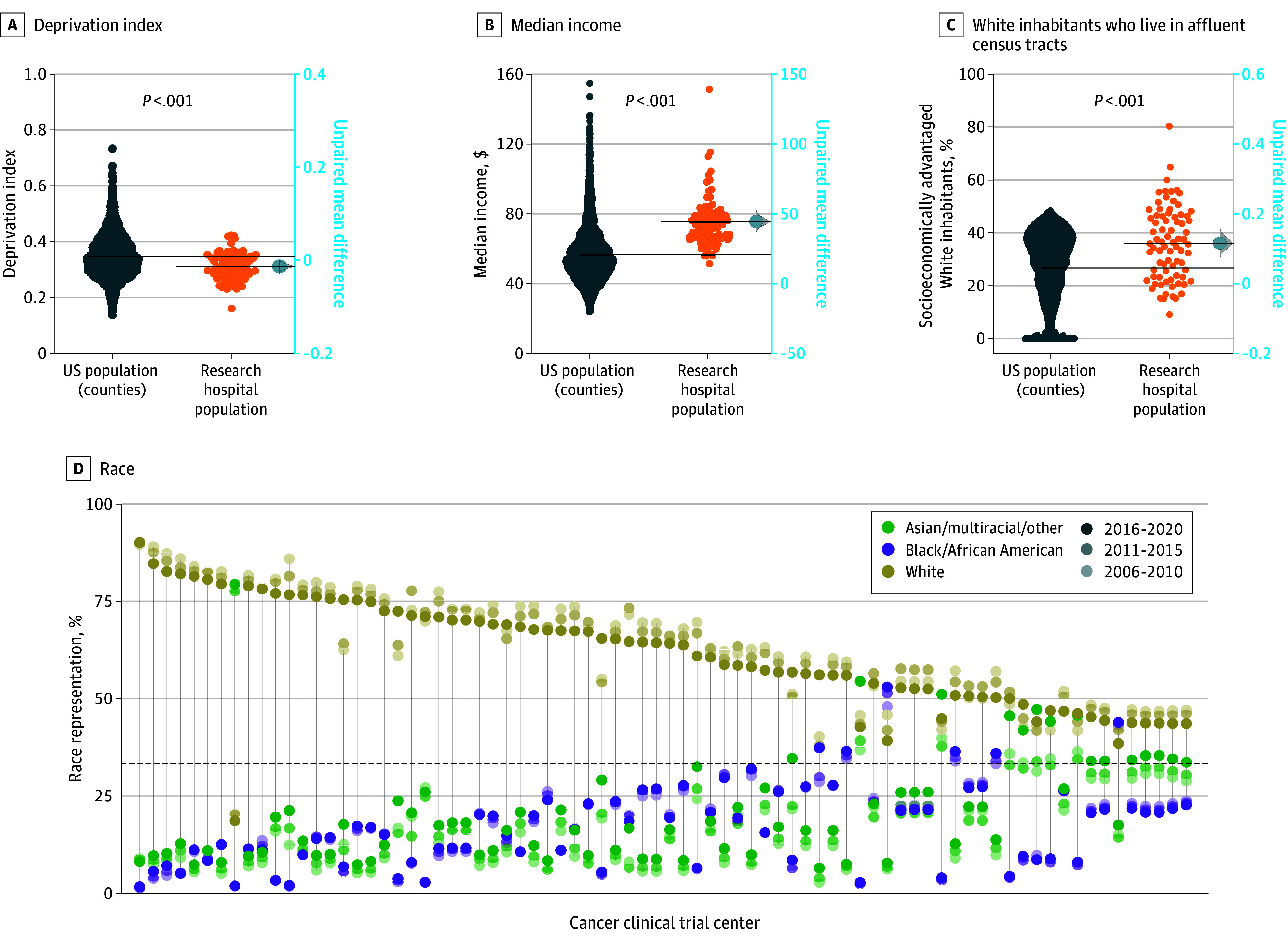
Characterization of the 78 Most Active US Cancer Clinical Trial Center Catchment Populations A, Swarmplot showing the deprivation index for each cancer clinical trial hospital catchment population and for each US county population. Deprivation index values closer to 1 represent greater social deprivation. Unpaired mean difference and 95% CI with bootstrap resampling shown on right axis (−0.035 unpaired mean difference; 95% CI, −0.047 to −0.024; *P* value calculated with Wilcoxon test). B, Swarmplot showing the median income for each cancer clinical trial hospital catchment population and for each US county. Unpaired mean difference and 95% CI with bootstrap resampling shown on right axis (+$18 900 unpaired mean difference; 95% CI, +$15 700 to +$22 400; *P* value calculated with Wilcoxon test). C, Swarmplot showing the percentage of inhabitants that both self-identify as White and live in census tracts that are more affluent than the national median (deprivation index below the national median) for each included cancer trial site catchment population or each US county. Unpaired mean difference and 95% CI with bootstrap resampling shown on right axis (+10.1% unpaired mean difference; 95% CI, +6.8% to +13.7%; *P* value calculated with Wilcoxon test). D, Dot plot showing the percentage representation of the 3 race categories with lines connecting each research site. Sites are ordered by descending order of percentage representation of the majority race group of each trial center catchment population. We show percentage representation between 2016 and 2020 (100% opacity), 2011 and 2015 (50% opacity), 2006 and 2010 (25% opacity).

This study did not require institutional review board approval nor was patient consent required, as it did not use any identifiable, confidential, or patient-level data. The volume of major US clinical cancer trial sites was queried on the national registry (eMethods, eFigure 1, eTable 1, and eTable 2 in Supplement 1). Catchment populations living within simulated driving distances^6^ from these sites were identified (eMethods in Supplement 1) and compared with the US general population (Figure 1A-C). Extended time-based analyses were calculated on the American Community Survey (ACS) 5-year survey data for 2006 to 2010, 2011 to 2015, and 2016 to 2020 (eMethods in Supplement 1).

Catchment-level socioeconomic deprivation indices^7^ and median income values were calculated as population weighted means of census tract-level data from the 2020 ACS. Catchment-level racial representation was calculated by combining the reported tract-level population counts from the 2020 census (eMethods in Supplement 1). Two-group estimation graphs were plotted with a false positive rate for significance set at *P* < .05.

We performed sensitivity analyses of the catchment populations within 30-, 60-, and 120-minute 1-way driving times from all US hospitals (N = 7623; eMethods in Supplement 1) based on published time cutoffs for trial commutes in Eastern urban sites and more rural suburban sites in the Midwest and West. Catchments with population sizes estimated to be large enough to recruit for phase 1, phase 2, and phase 3 trials were selected using national mean trial participation rates (eMethods in Supplement 1). We then filtered for the top 20th percentile and 50th percentile of locations rank-ordered by the diversity score of their catchment populations. Statistical analyses were performed on data collected between 2006 and 2020 using R version 4.1.3 (R Project for Statistical Computing).

## Results

Populations living within the 30-minute commute catchment area around the 78 major US cancer research centers were composed of more affluent census tracts with lower deprivation indices compared with the mean US county population (−0.035 unpaired mean difference; 95% CI, −0.047 to −0.024; Figure 1A). The median income of the catchment populations was also significantly greater (+$18 900 unpaired mean difference; 95% CI, +$15 700 to +$22 400; Figure 1B). Overall, the cancer clinical trial sites had a higher proportion of White inhabitants living in affluent tracts compared with the mean US county population (+10.1% unpaired mean difference; 95% CI, +6.8% to +13.7%; Figure 1C).

Some of the 78 cancer trial sites were surrounded with approximately equal representation of the 3 racial groups (Asian/multiracial/other [Asian alone, American Indian or Alaska Native alone, Native Hawaiian or Other Pacific Islander alone, some other race alone, population of 2 or more races], Black or African American, White), whereas others approached minoritized population representation of 2% to 3%. A single group comprised more than half of the catchment inhabitants for 65 centers (Asian/multiracial/other [n = 3], Black [n = 1], White [n = 61]) (Figure 1D). Visualization of corresponding self-identified race data from historical datasets showed increasing catchment area diversity in recent quinquennia.

**Figure 2.**
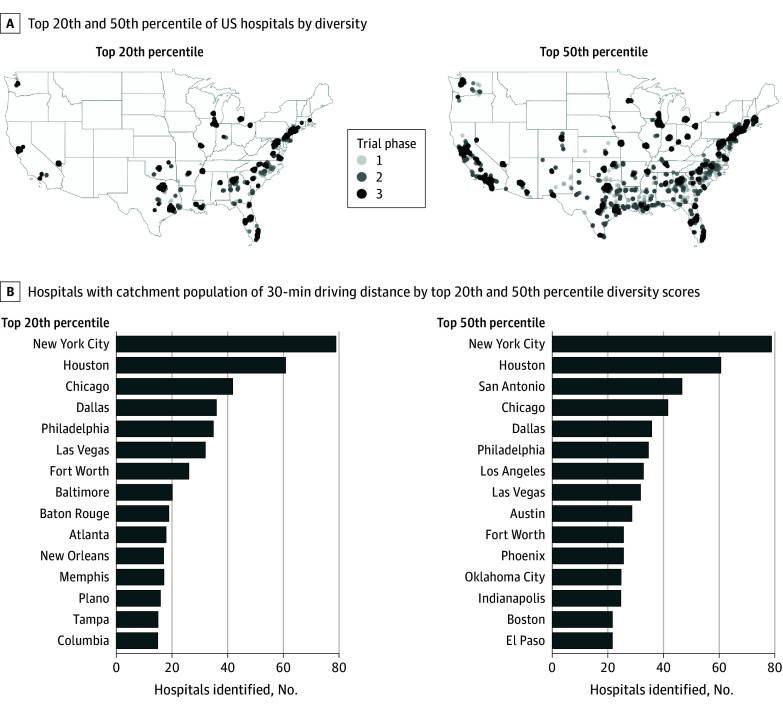
Identification of New Locations With Equitable Commuting Access for Racially Diverse Catchment Populations A, Sensitivity analysis showing locations of the top 20th percentile and top 50th percentile of US hospitals ranked by the diversity scores of their catchment populations (defined as inhabitants living in tracts within 30-minute 1-way drive) and has sufficient population size to enroll patients with cancer for phase 1, 2, or 3 trials. B, Bar graph showing the 15 cities with the highest number of hospitals identified as having a catchment population of 30-minute 1-way driving distance with top 20th percentile or 50th percentile diversity scores.

To identify existing hospital sites geographically closest to the most racially diverse populations, we performed a sensitivity analysis of the catchment populations of all national US hospitals within 30-, 60-, or 120-minute 1-way driving commute time boundaries (Figure 2, eFigure 2 in Supplement 1).

**Figure 3.**
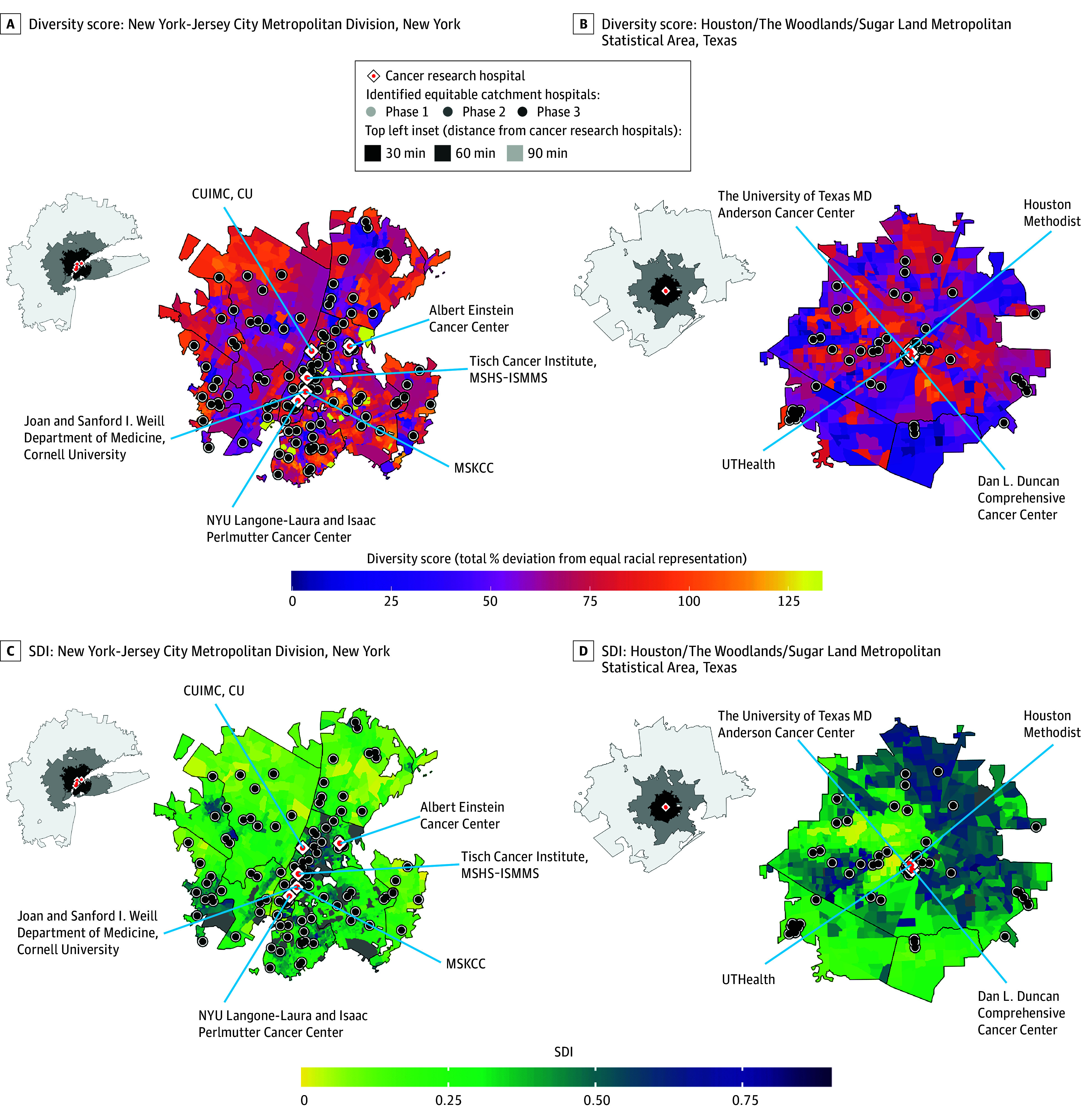
Mapped Visualization of Diversity Scores and Socioeconomic Deprivation Indices With Overlay of Potential New Trial Site Locations and Commute Boundaries From Existing Cancer Clinical Trial Centers A, New York-Jersey City Metropolitan division map of commuting-populations within 30-minute travel time to existing cancer research hospitals. Top left inset outline the areas that fall under 120-, 60-, or 30-minute commute times from the cancer research hospitals within the city. Detailed commuting populations for all cities shown in eFigure 3 in Supplement 1. Tracts are colored by diversity scores (with darker-colored purple tracts showing greatest racial diversity with closest to equal representation of one-third Asian/multiracial/other [Asian alone, American Indian or Alaska Native alone, Native Hawaiian or Other Pacific Islander alone, some other race alone, population of 2 or more races], one-third Black or African American, and one-third White populations). Existing hospitals identified through sensitivity analyses as ideally located for equal racial representation (within the top 20th percentile, for catchment populations within 30-minute 1-way driving distance) are shown in increasingly dark shades of gray for phase 1, 2, and 3 trials, respectively. Otherwise preexisting hospitals are shown in white. B, Houston/The Woodlands/Sugar Land Metropolitan Statistical Area with tracts colored by diversity scores and with same overlays as in panel A. C, New York-Jersey City Metropolitan division map with tracts colored by census level socioeconomic deprivation indices. Deprivation index values closer to 1 represent greater social deprivation. Darker-colored tracts with values closer to 1 represent greater socioeconomic deprivation and same overlays as panel A. D, Houston/The Woodlands/Sugar Land Metropolitan Statistical Area map with tracts colored by socioeconomic deprivation indices and same overlays as panel A.

To visualize and identify the diverse census tracts within close proximity to existing cancer clinical trial sites and/or located in densely populated areas, we drew maps colored by racial diversity scores and deprivation indices with overlays of existing hospital sites, major US cancer clinical research sites, and commuting distances to the closest major US cancer clinical research sites. Example maps of New York and Houston, which consistently ranked as the top 2 cities with the most hospitals with the greatest diversity of catchment populations (Figure 2, eFigure 2 in Supplement 1), are displayed in Figure 3. A complete atlas of all cities with a population above 500 000, as well as all cities with cancer clinical trial centers are shown in eFigure 3 in Supplement 1.

These maps highlight hospitals within cities that exist within or near urban areas with high racially and socioeconomically diverse populations and are also located close to existing cancer research hospitals that have the infrastructure in place to conduct cancer clinical trials. Most urban areas in the US have hospitals located in socioeconomically disadvantaged and racially diverse areas, areas that frequently colocalize.^8,9^

## Discussion

This study indicates that geographical population distributions may present barriers to equitable clinical trial access and that data are available to proactively strategize about reduction of such barriers. In addition, our findings draw attention to modifiable socioeconomic factors, such as affordable transportation and increased financial toxicity related to trial participation.^10,11^ Many populations excluded from trial participation—minoritized racial and ethnic groups, young adults, older adults, rural patients, and sexual/gender minority groups experience the greatest social risk factors,^5^ including lower socioeconomic status, a key mediator of reduced trial participation.^12,13^ Geography further limits access to trials^6,14^ and may compound the socioeconomic burdens associated with clinical trial participation (eg, time to travel and logistical problems).^15^ As most clinical trials require additional hospital visits, probability of trial participation decreases as travel time increases.^6^ After controlling for income, racial and ethnic groups have consented to trials at the same rates when they are offered, showing the importance of reducing structural barriers to address unequal participation.^16^

Apart from providing a detailed reference for the current hospital, cancer clinical trial center, and population distribution, we hope our work will assist in many efforts. For example, our analyses can aid studies that aim to determine the effect size of location on trial enrollment inequity. Existing clinical trial centers may build collaborative efforts with nearby hospitals closer to underrepresented populations or set up community centers to support new collaborative networks to improve geographical access equity. Methodologically, our approach is transferrable to any country, region, or global effort with sufficient source data and can inform decision-making along the continuum of cancer care, from screening to implementing specialist care.

### Limitations

This study has limitations. The chosen threshold values for travel time and diversity are proof-of-concept examples. The calculated catchment areas may differ from the true populations served by a cancer center.^17^ Satellite sites and weighted enrollment are not included in our analyses. Although we use the national mean trial participation rate for our nationwide sensitivity analyses, individual institutions will have differing trial participation rates. Also, the participation rates would be higher if all clinical research studies and not just interventional trials were considered. We provide open access to the analytical code to facilitate customized adjustments. Access to public or private transportation can present participation barriers for patients and the cost other than time investment can present a further limitation that our study does not quantify or simulate.

Individuals who are American Indian or Alaska Native, Pacific Islander, or from rural populations face specific challenges because of lower population size and density, which may require decentralized clinical trials for greater inclusion. Hispanic ethnicity overlaps with the other population categories and requires separate analysis. Additionally, the national trial registry only approximates trial center activity.

## Conclusions

This study found that populations in the proximity of high-activity US clinical trial centers are less diverse, and potential trial enrollment sites with highly diverse populations can be identified using available data. Data-driven approaches may reduce current disparities in clinical trial populations.

## Notes

Supplement 1. **eFigure 1.** Cancer Clinical Trial Activity From U.S.-Based Trial Sites Between 2012–2022 **eFigure 2.** Expanded Sensitivity Analysis for Identifying New Locations With Equitable Commuting Access for Diverse Catchment Populations **eFigure 3.** Maps of All U.S. Cities With a Population Above 500K or With Existing Cancer Clinical Trial Centers Showing Potential New Trial Site Locations, Commuting Boundaries From Existing Cancer Clinical Trial Centers, and Racial Representation or Deprivation Scores **eTable 1.** Quantitative Summary of U.S. Cancer Clinical Trial Sites **eTable 2.** List and Quantitative Summary of Individual U.S. Cancer Clinical Trial Sites in Alphabetical Order **eMethods.** Unabridged Data Sources, Calculations, and Analysis
eReferences

Supplement 2.
Data Sharing Statement

## References

[1] The National Commission for the Protection of Human Subjects of Biomedical and Behavioral Research. The Belmont Report. Published 1979. Accessed February 13, 2024. https://www.hhs.gov/ohrp/regulations-and-policy/belmont-report/index.html

[2] FDA. FDA takes important steps to increase racial and ethnic diversity in clinical trials. Accessed June 16, 2022. https://www.fda.gov/news-events/press-announcements/fda-takes-important-steps-increase-racial-and-ethnic-diversity-clinical-trials

[3] Winn RA. Enrollment matters: the reality of disparity and pursuit of equity in clinical trials. Cancer Discov. 2022;12(6):1419-1422. doi:10.1158/2159-8290.CD-22-0319 35652212

[4] Dizon DS, Krilov L, Cohen E, . Clinical Cancer Advances 2016: annual report on progress against cancer from the American Society of Clinical Oncology. J Clin Oncol. 2016;34(9):987-1011. doi:10.1200/JCO.2015.65.8427 26846975 PMC5075244

[5] Unger JM, Vaidya R, Hershman DL, Minasian LM, Fleury ME. Systematic review and meta-analysis of the magnitude of structural, clinical, and physician and patient barriers to cancer clinical trial participation. J Natl Cancer Inst. 2019;111(3):245-255. doi:10.1093/jnci/djy221 30856272 PMC6410951

[6] Legge F, Eaton D, Molife R, . Participation of patients with gynecological cancer in phase I clinical trials: two years experience in a major cancer center. Gynecol Oncol. 2007;104(3):551-556. doi:10.1016/j.ygyno.2006.09.020 17064758

[7] Brokamp C, Beck AF, Goyal NK, Ryan P, Greenberg JM, Hall ES. Material community deprivation and hospital utilization during the first year of life: an urban population-based cohort study. Ann Epidemiol. 2019;30:37-43. doi:10.1016/j.annepidem.2018.11.008 30563729 PMC6370517

[8] Williams DR, Priest N, Anderson NB. Understanding associations among race, socioeconomic status, and health: Patterns and prospects. Health Psychol. 2016;35(4):407-411. doi:10.1037/hea0000242 27018733 PMC4817358

[9] DeNavas-Walt C, Proctor BD. Income and poverty in the United States: 2013. United States Census Bureau. Published online September 16, 2014. Accessed February 28, 2024. https://www.census.gov/library/publications/2014/demo/p60-249.html

[10] Winkfield KM, Phillips JK, Joffe S, Halpern MT, Wollins DS, Moy B. Addressing financial barriers to patient participation in clinical trials: ASCO policy statement. J Clin Oncol. 2018;36(33):JCO1801132. doi:10.1200/JCO.18.01132 30212297

[11] Chino F, Zafar SY. Financial toxicity and equitable access to clinical trials. Am Soc Clin Oncol Educ Book. 2019;39(39):11-18. doi:10.1200/EDBK_100019 31099681

[12] Sharrocks K, Spicer J, Camidge DR, Papa S. The impact of socioeconomic status on access to cancer clinical trials. Br J Cancer. 2014;111(9):1684-1687. doi:10.1038/bjc.2014.108 25093493 PMC4453719

[13] Unger JM, Hershman DL, Albain KS, . Patient income level and cancer clinical trial participation. J Clin Oncol. 2013;31(5):536-542. doi:10.1200/JCO.2012.45.4553 23295802 PMC3565180

[14] Levit LA, Byatt L, Lyss AP, . Closing the rural cancer care gap: three institutional approaches. JCO Oncol Pract. 2020;16(7):422-430. doi:10.1200/OP.20.00174 32574128

[15] Borno HT, Zhang L, Siegel A, Chang E, Ryan CJ. At what cost to clinical trial enrollment? a retrospective study of patient travel burden in cancer clinical trials. Oncologist. 2018;23(10):1242-1249. doi:10.1634/theoncologist.2017-0628 29700209 PMC6263122

[16] Unger JM, Hershman DL, Till C, . “When Offered to Participate”: a systematic review and meta-analysis of patient agreement to participate in cancer clinical trials. J Natl Cancer Inst. 2021;113(3):244-257. doi:10.1093/jnci/djaa155 33022716 PMC7936064

[17] Leader AE, McNair C, Yurick C, . Assessing the Coverage of US Cancer Center Primary Catchment Areas. Cancer Epidemiol Biomarkers Prev. 2022;31(5):955-964. doi:10.1158/1055-9965.EPI-21-1097 35064067 PMC9081121

