# Supplementary material for: Analysis and Optimization of Equitable US Cancer Clinical Trial Center Access by Travel Time
Source: JAMA Oncol. 2024 Mar 21;10(5):652–7. doi: 10.1001/jamaoncol.2023.7314 (PMC10958387; doi:10.1001/jamaoncol.2023.7314)
Supplement: Supplement 1. — eFigure 1. Cancer Clinical Trial Activity From U.S.-Based Trial Sites Between 2012–2022 eFigure 2. Expanded Sensitivity Analysis for Identifying New Locations With Equitable Commuting Access for Diverse Catchment Populations eFigure 3. Maps of All U.S. Cities With a Population Above 500K or With Existing Cancer Clinical Trial Centers Showing Potential New Trial Site Locations, Commuting Boundaries From Existing Cancer Clinical Trial Centers, and Racial Representation or Deprivation Scores eTable 1. Quantitative Summary of U.S. Cancer Clinical Trial Sites eTable 2. List and Quantitative Summary of Individual U.S. Cancer Clinical Trial Sites in Alphabetical Order eMethods. Unabridged Data Sources, Calculations, and Analysis eReferences [file jamaoncol-e237314-s001.pdf]

Supplemental Online Content

Lee H, Bates AS, Callier S, et al. Analysis and Optimization of Equitable US Cancer Clinical Trial Center Access by Travel Time. *JAMA Oncol.* Published online March 21, 2024. doi:10.1001/jamaoncol.2023.7314

eFigure 1. Cancer Clinical Trial Activity From U.S.-Based Trial Sites Between 2012–2022

eFigure 2. Expanded Sensitivity Analysis for Identifying New Locations With Equitable Commuting Access for Diverse Catchment Populations

eFigure 3. Maps of All U.S. Cities With a Population Above 500K or With Existing Cancer Clinical Trial Centers Showing Potential New Trial Site Locations, Commuting Boundaries From Existing Cancer Clinical Trial Centers, and Racial Representation or Deprivation Scores

eTable 1. Quantitative Summary of U.S. Cancer Clinical Trial Sites

eTable 2. List and Quantitative Summary of Individual U.S. Cancer Clinical Trial Sites in Alphabetical Order

eMethods. Unabridged Data Sources, Calculations, and Analysis

eReferences

This supplemental material has been provided by the authors to give readers additional information about their work.

© 2024 Lee H et al. *JAMA Oncology.*

eFigure 1. Cancer clinical trial activity from U.S.-based trial sites between 2012-2022

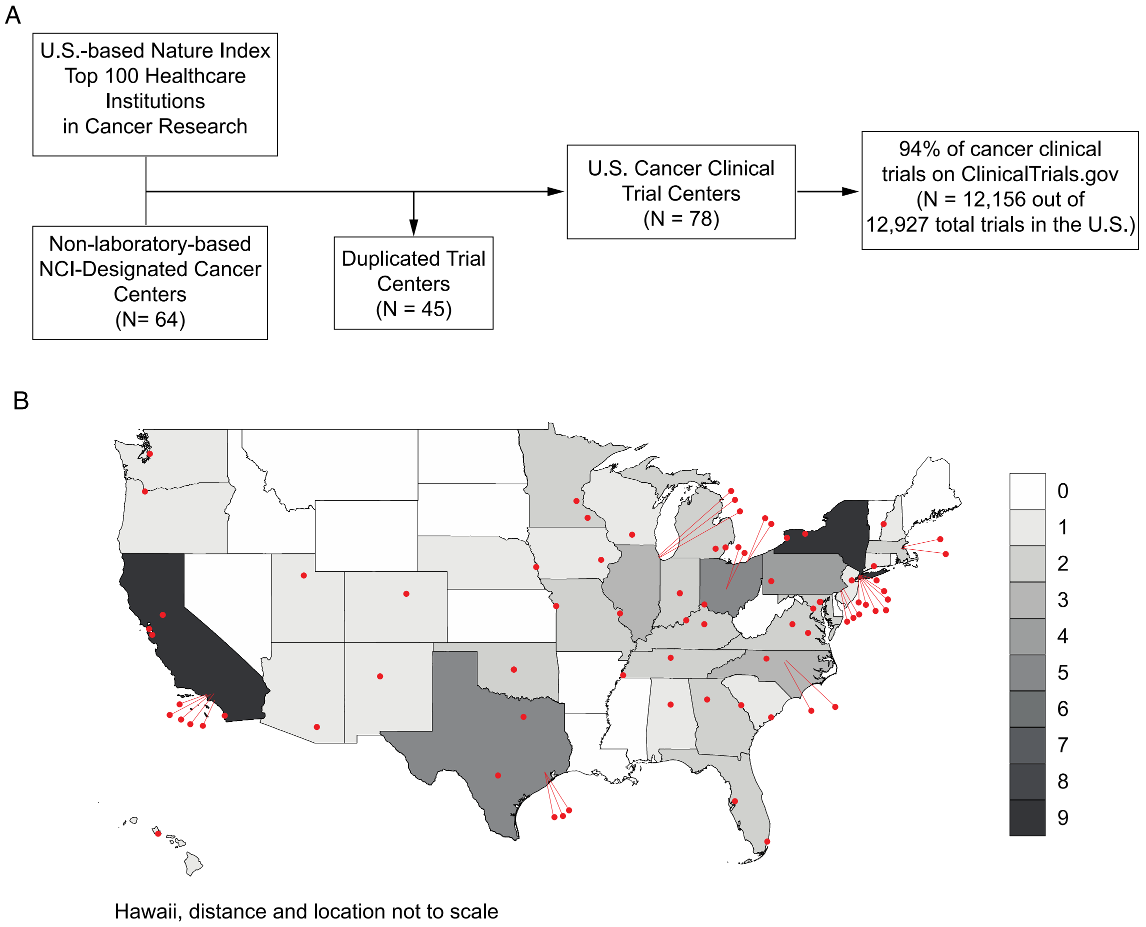

A) Schematic of workflow for clinical trial center identification and quantitative assessment. We queried the U.S. national trial registry (nct.gov) to quantify the total number of adult phase 1–3 cancer clinical trials between 2012–2022 from our list of research sites. The search was filtered by age (>18), location (U.S.), trial type (phase 1, 2, or 3) and recruitment status (excluded if ‘terminated’ or ‘withdrawn’) or if the trial had more hospital sites registered than total number of patients targeted for enrollment. B) Location and state density of the 78 most active cancer clinical trial sites by total number of trials.

© 2024 Lee H et al. *JAMA Oncology.*

eFigure 2. Expanded sensitivity analysis for identifying new locations with equitable commuting access for racially diverse catchment populations

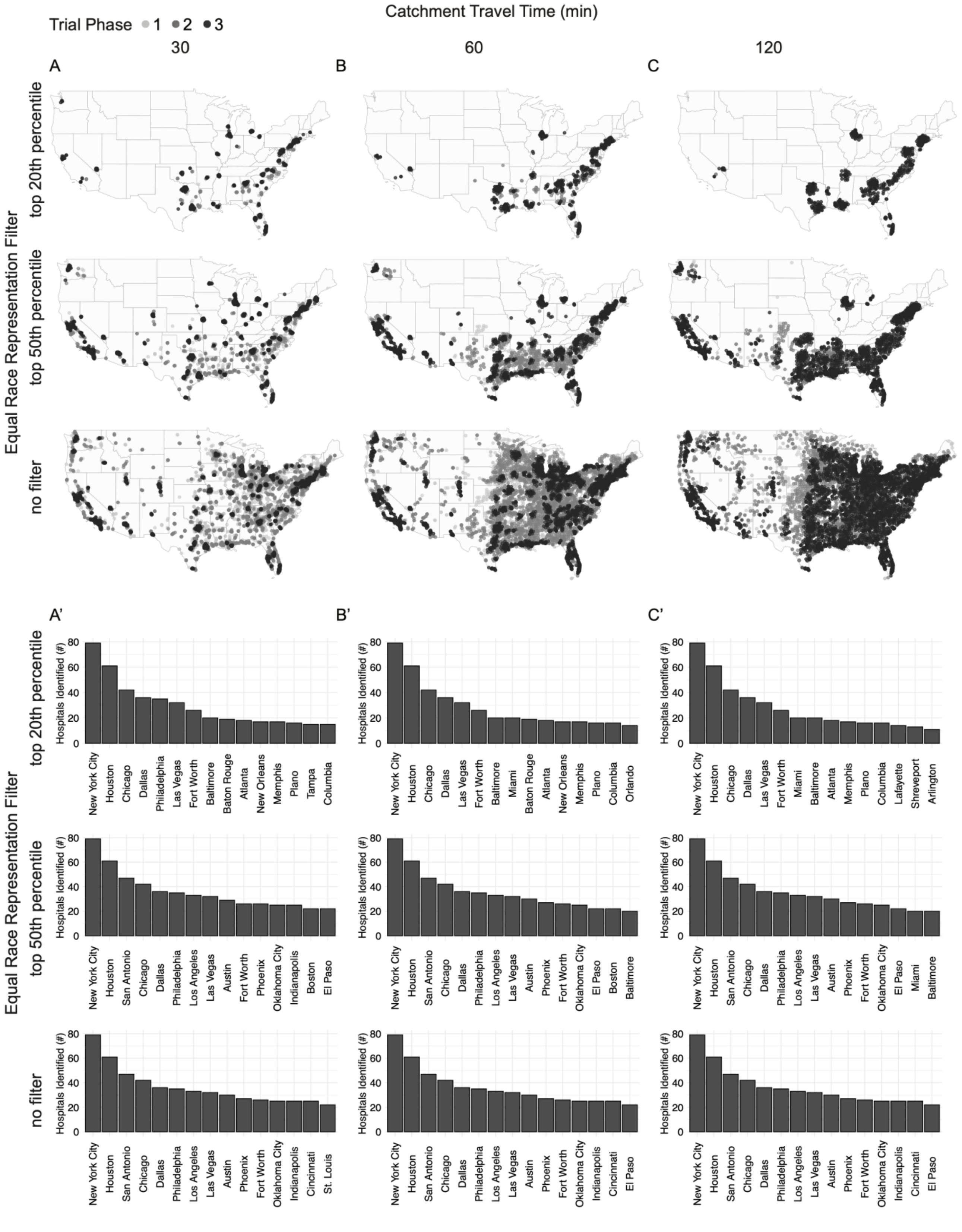

© 2024 Lee H et al. *JAMA Oncology.*

Location of the top 20th and 50th percentile of U.S. hospitals ranked by their diversity score (total percentage deviation from equal representation of 1/3rd White, 1/3rd Black/African American, 1/3rd Asian/Mixed/Other groups) for catchment populations within a A) 30-, B) 60-, and C) 120-minute one-way drive. Catchment populations were also filtered for sufficient population size to facilitate enrolling patients with cancer for phase 1, 2, or 3 trials (light grey, dark grey, black, respectively). Results of sensitivity analyses can be compared with application of same population size filters with no applied diversity filter. Bar graphs showing the 15 cities with the highest number of hospitals identified as having a catchment population within the top 20th and 50th percentile for closeness to equal racial proportions for catchment populations within a A') 30-, B') 60-, and C') 120-minute one-way drive. Results of sensitivity analyses can be compared with application of same population size filters with no applied diversity filter.

© 2024 Lee H et al. *JAMA Oncology.*

eFigure 3. Maps of all U.S. cities with a population above 500K or with existing cancer clinical trial centers showing potential new trial site locations, commuting boundaries from existing cancer clinical trial centers, and racial representation or deprivation scores

From left to right, tracts are colored by the total % deviation from equal racial representation (33% White, 33% Black/African American, 33% Asian/Mixed/Other; proportionate racial representation (62% White, 12% Black/African American, 26% Asian/Mixed/Other); and deprivation index. Darker-colored tracts are those that are closest to desired racial representation by given scores. Cancer research hospitals are shown as a white vertical rhombus with a superimposed central red circle. Corresponding commute boundaries that fall under a 30-, 60-, or 120-minute one-way journey from those research sites are shown with striped black-white-black commute border lines with increasing transparency for increasing commuting time. Pre-existing other hospitals are shown in white (small circle). Existing hospitals identified as ideally located for equal racial representation (within the top 20th percentile, for catchment populations within 30-minute one-way driving distance) are shown in triple black-white-black bordered circles with increasing shades of grey (light grey, dark grey, black) for phase 1, 2, and 3 trials, respectively. Borders for each city are defined as core-based statistical areas (CBSA).

© 2024 Lee H et al. *JAMA Oncology.*

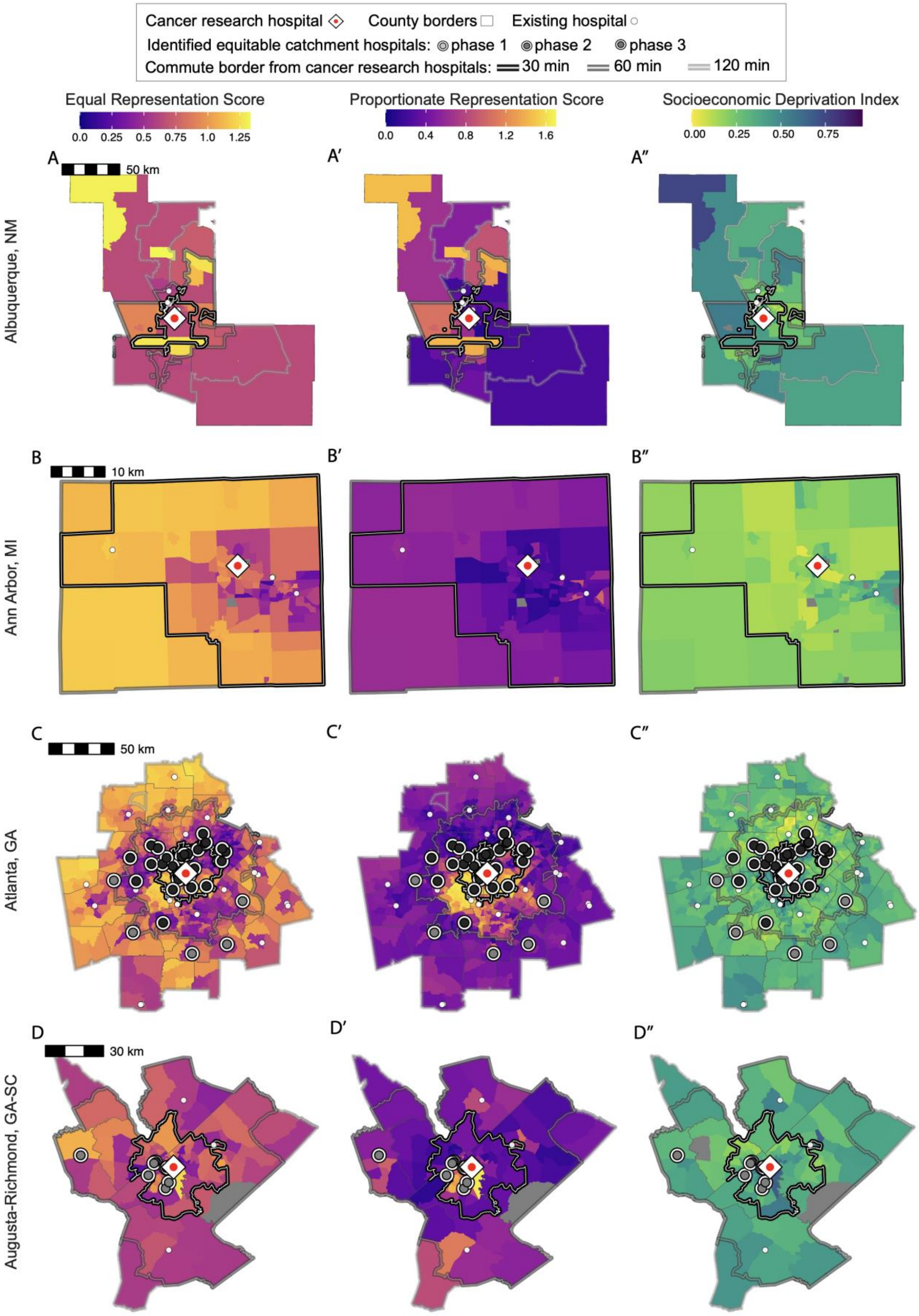

© 2024 Lee H et al. *JAMA Oncology.*

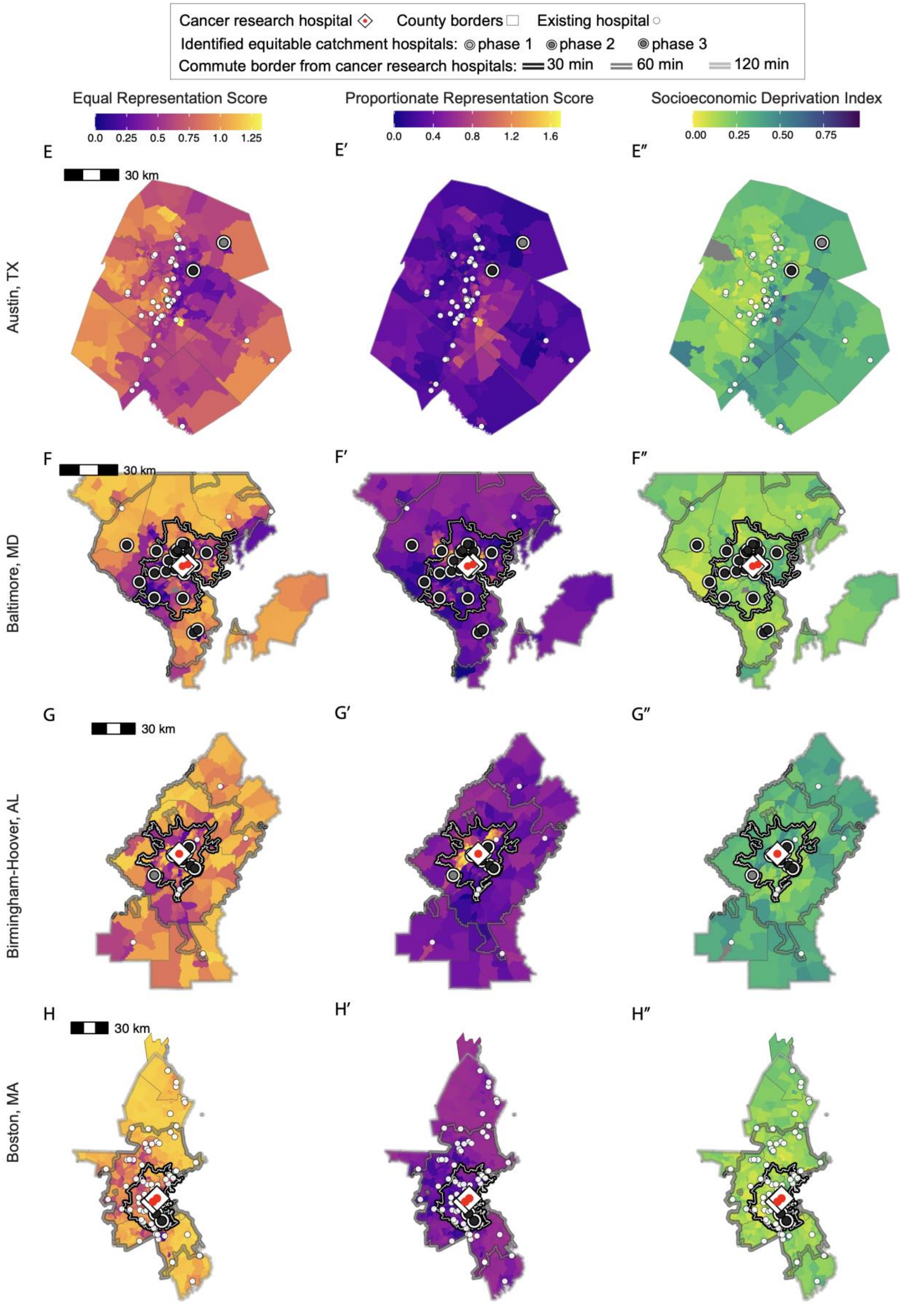

© 2024 Lee H et al. *JAMA Oncology*.

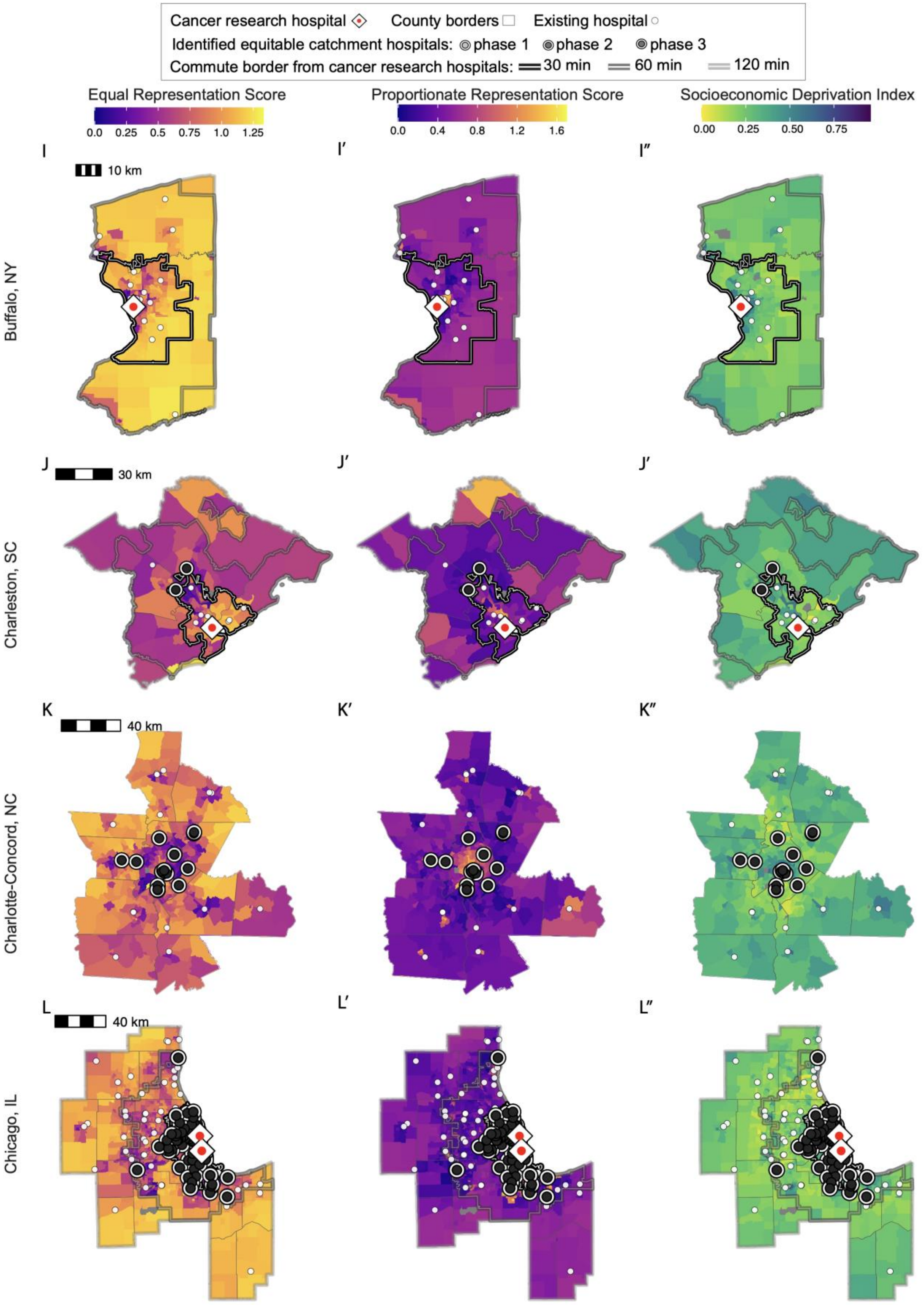

© 2024 Lee H et al. *JAMA Oncology.*

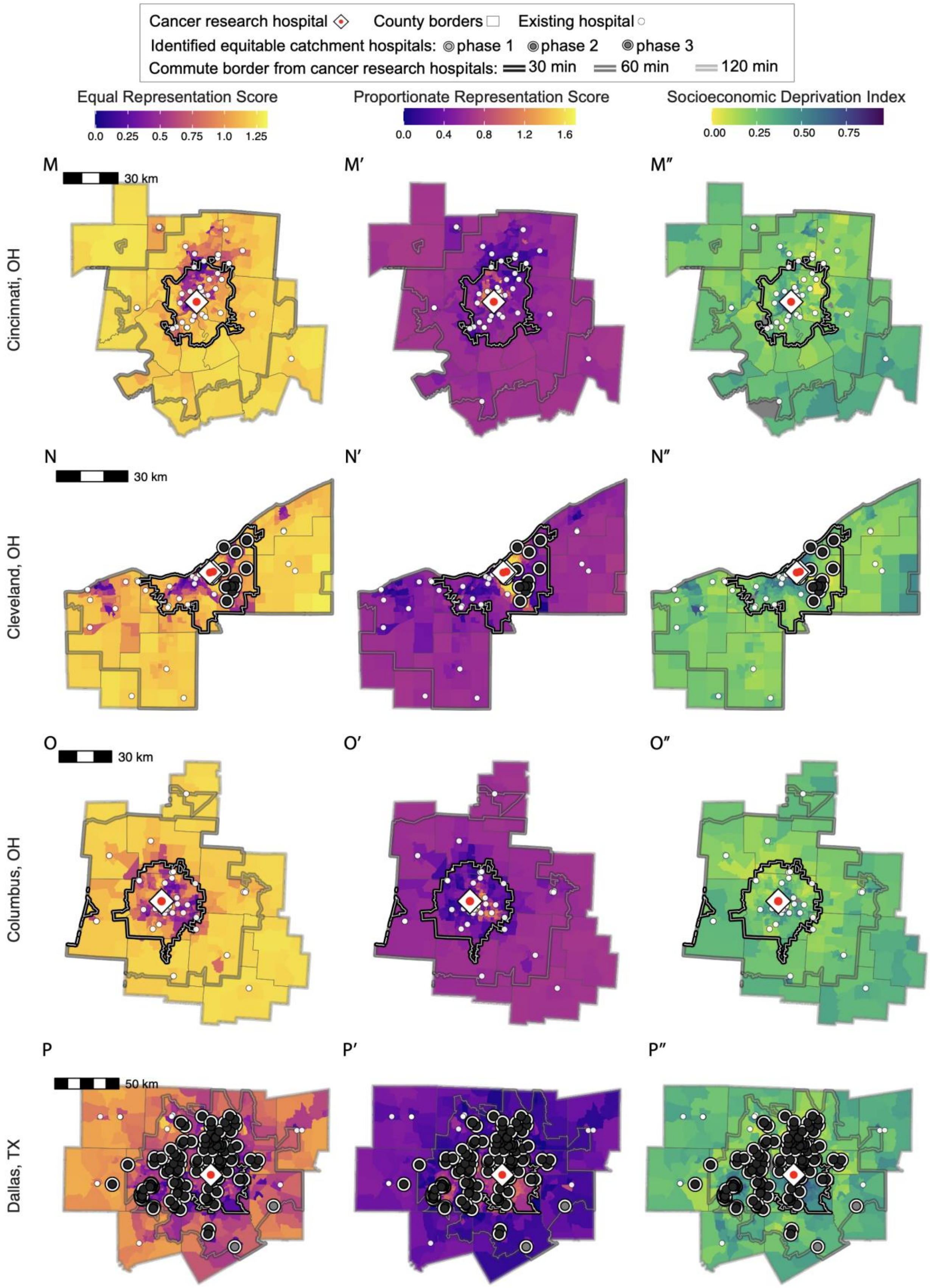

© 2024 Lee H et al. *JAMA Oncology.*

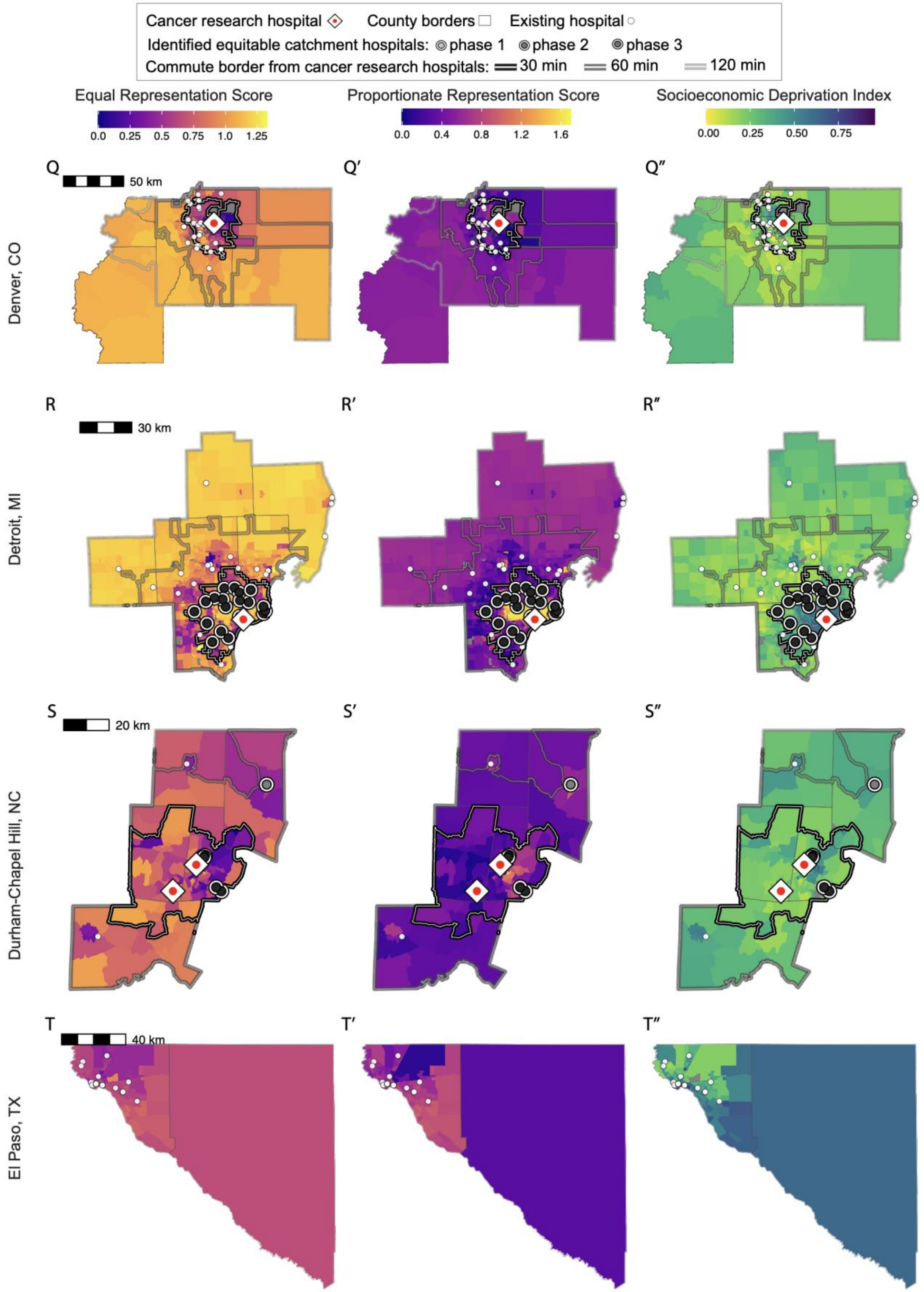

© 2024 Lee H et al. *JAMA Oncology.*

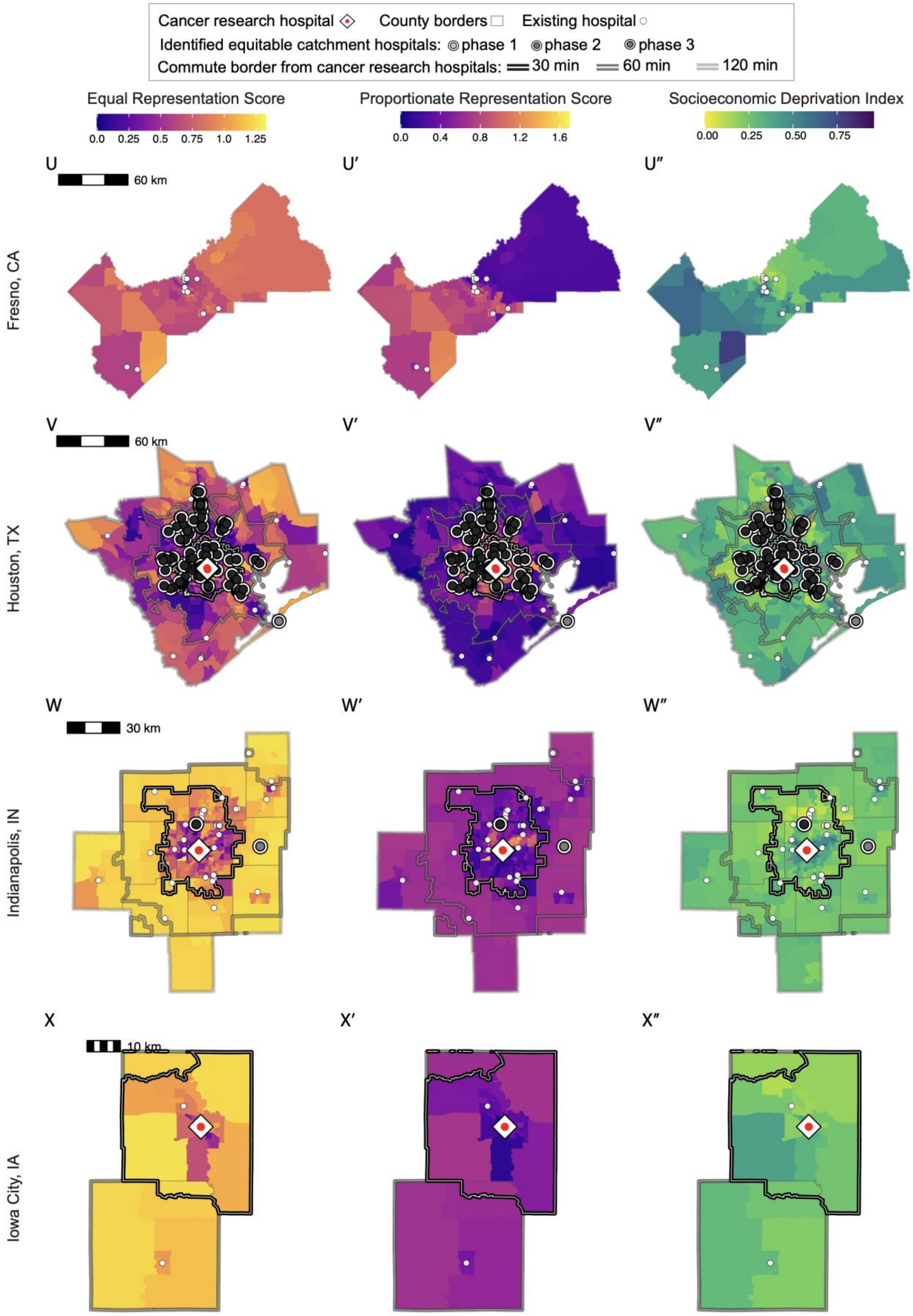

© 2024 Lee H et al. *JAMA Oncology.*

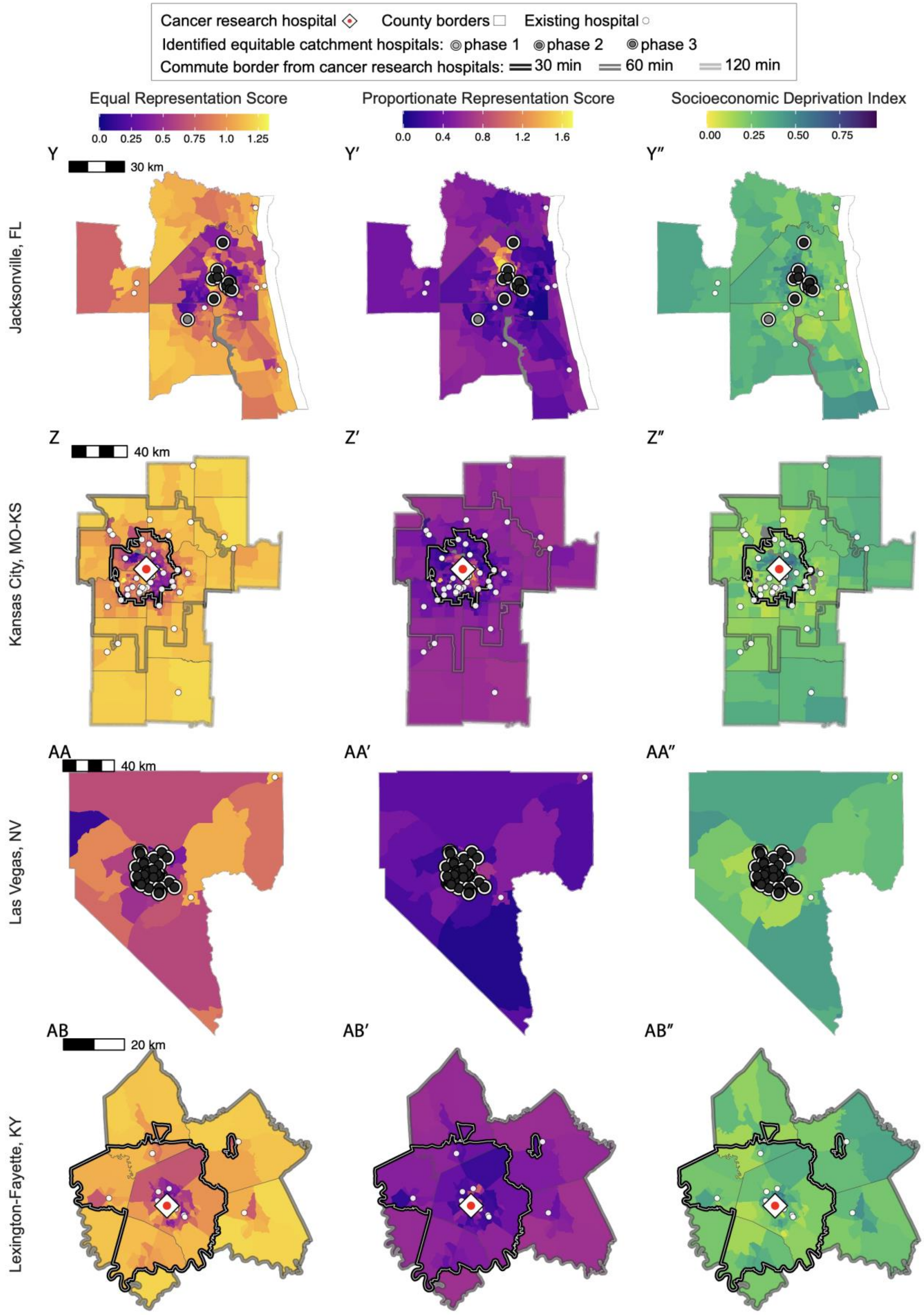

© 2024 Lee H et al. *JAMA Oncology.*

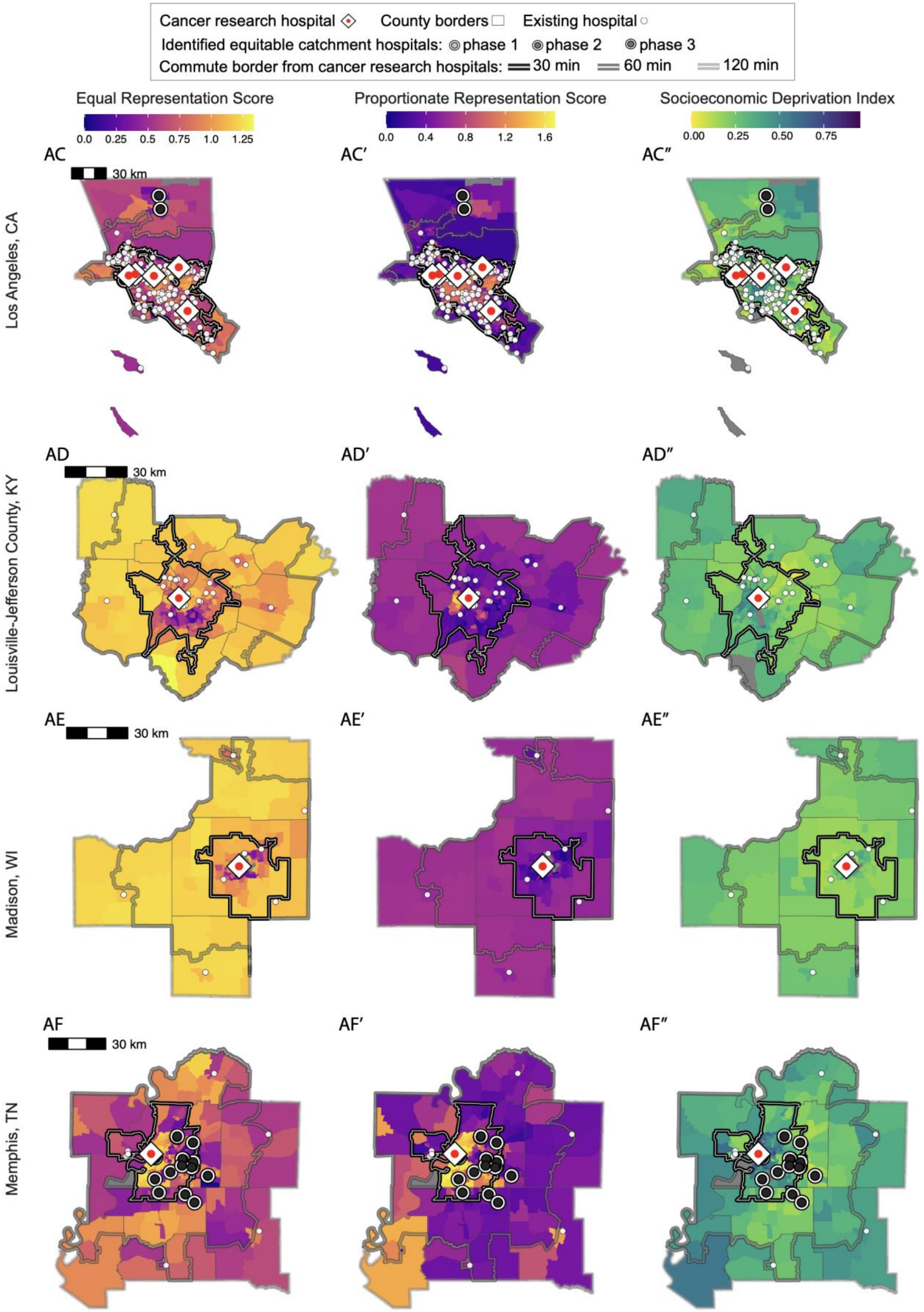

© 2024 Lee H et al. *JAMA Oncology.*

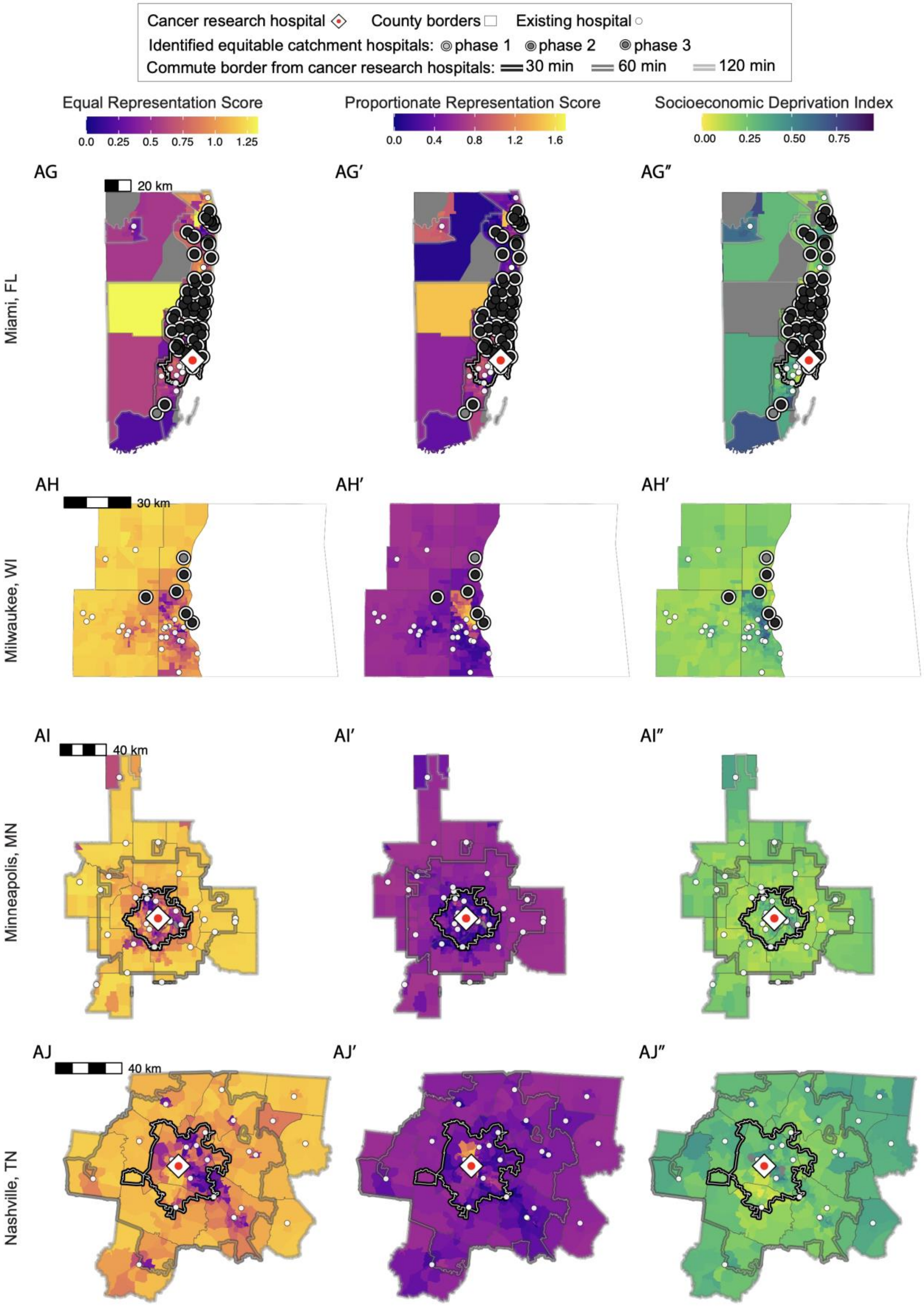

© 2024 Lee H et al. *JAMA Oncology.*

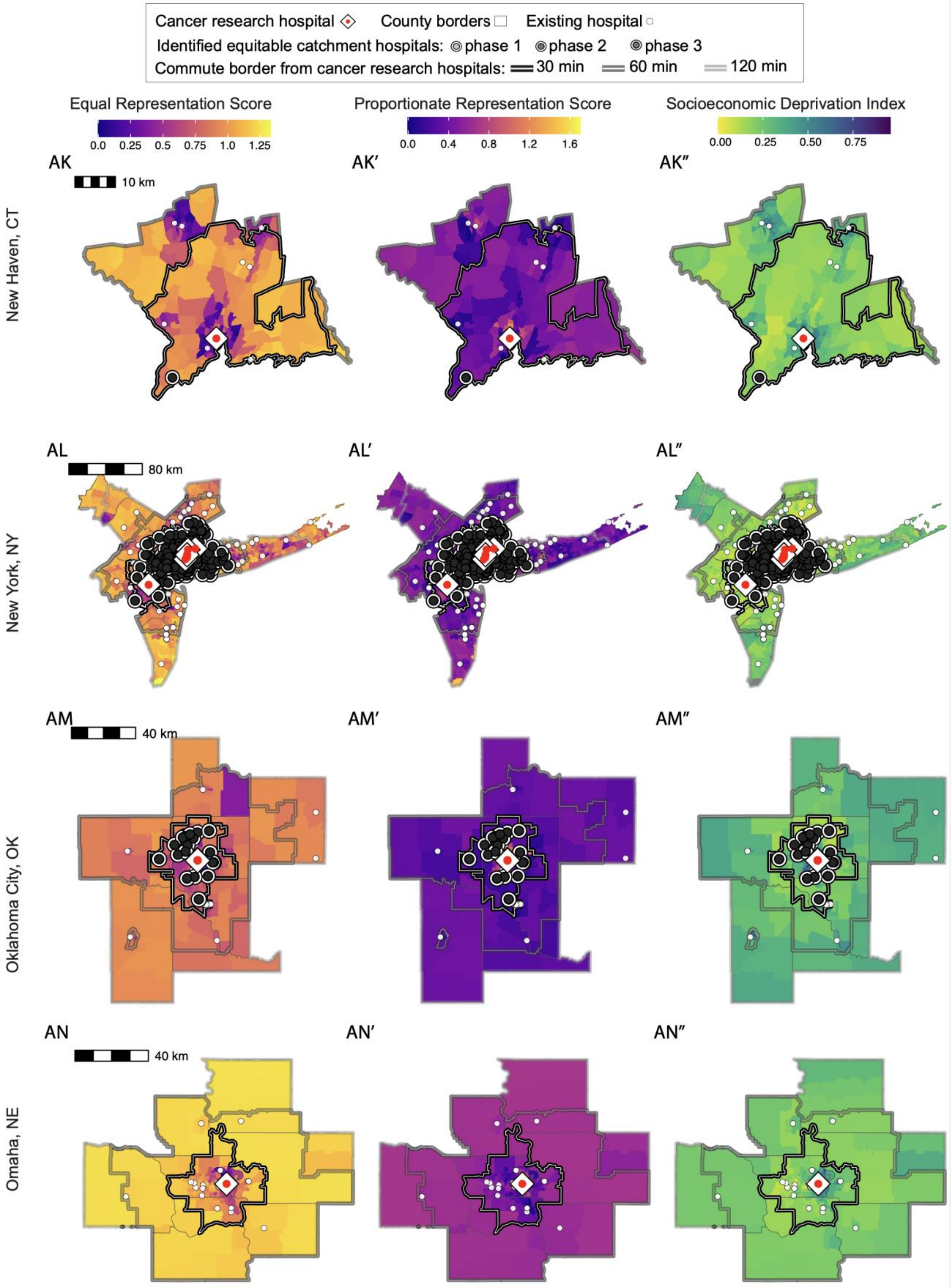

© 2024 Lee H et al. *JAMA Oncology.*

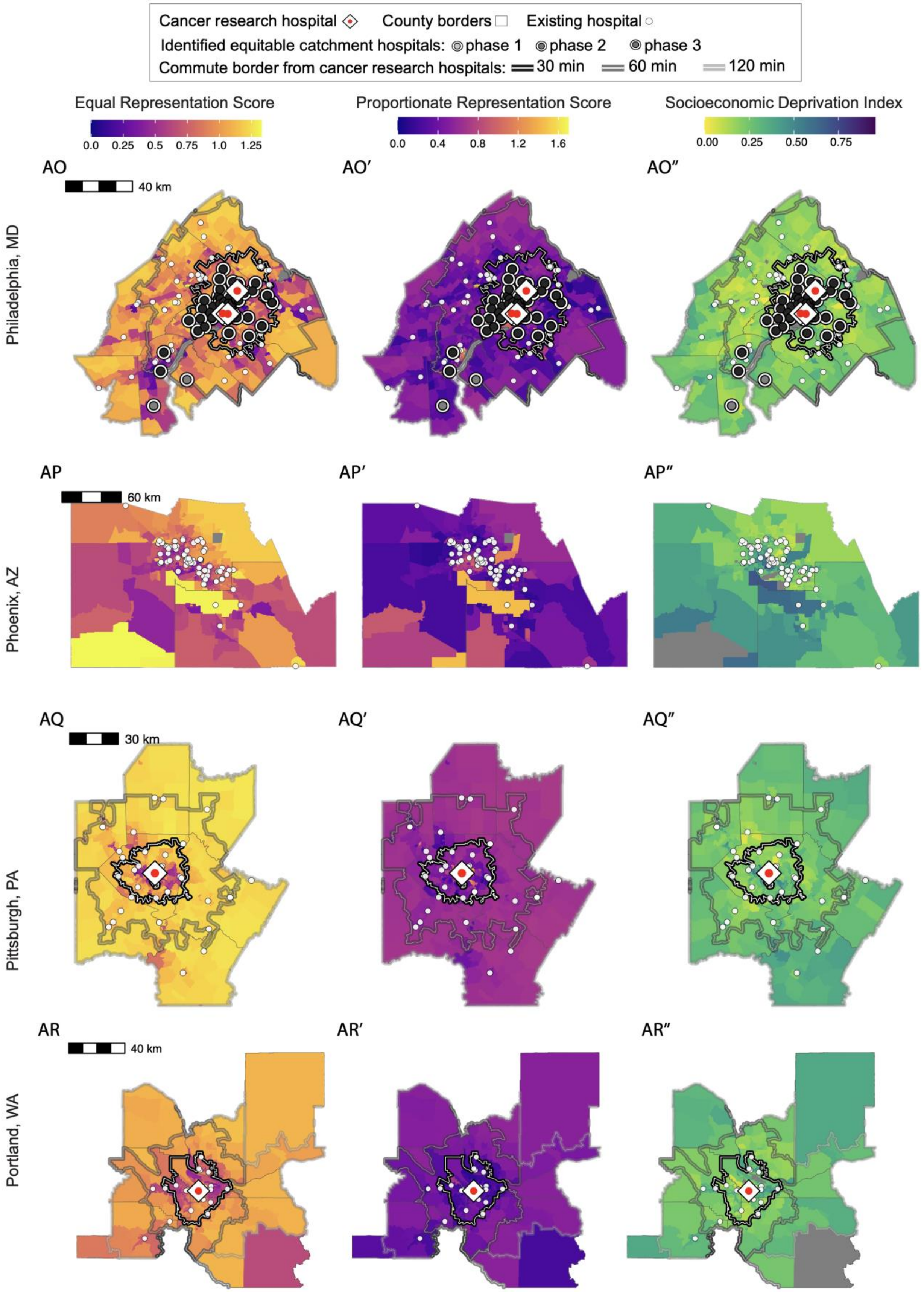

© 2024 Lee H et al. *JAMA Oncology.*

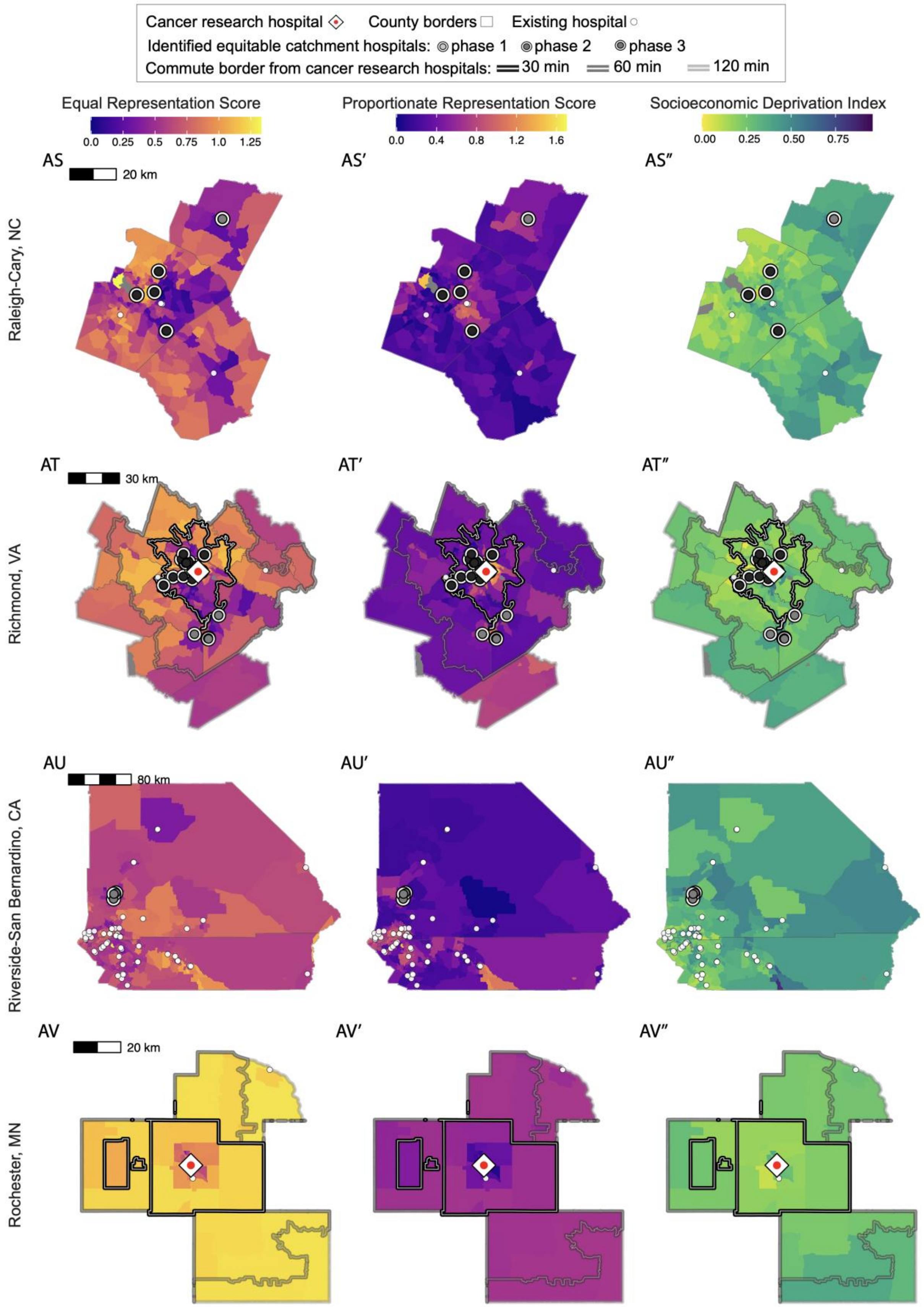

© 2024 Lee H et al. *JAMA Oncology.*

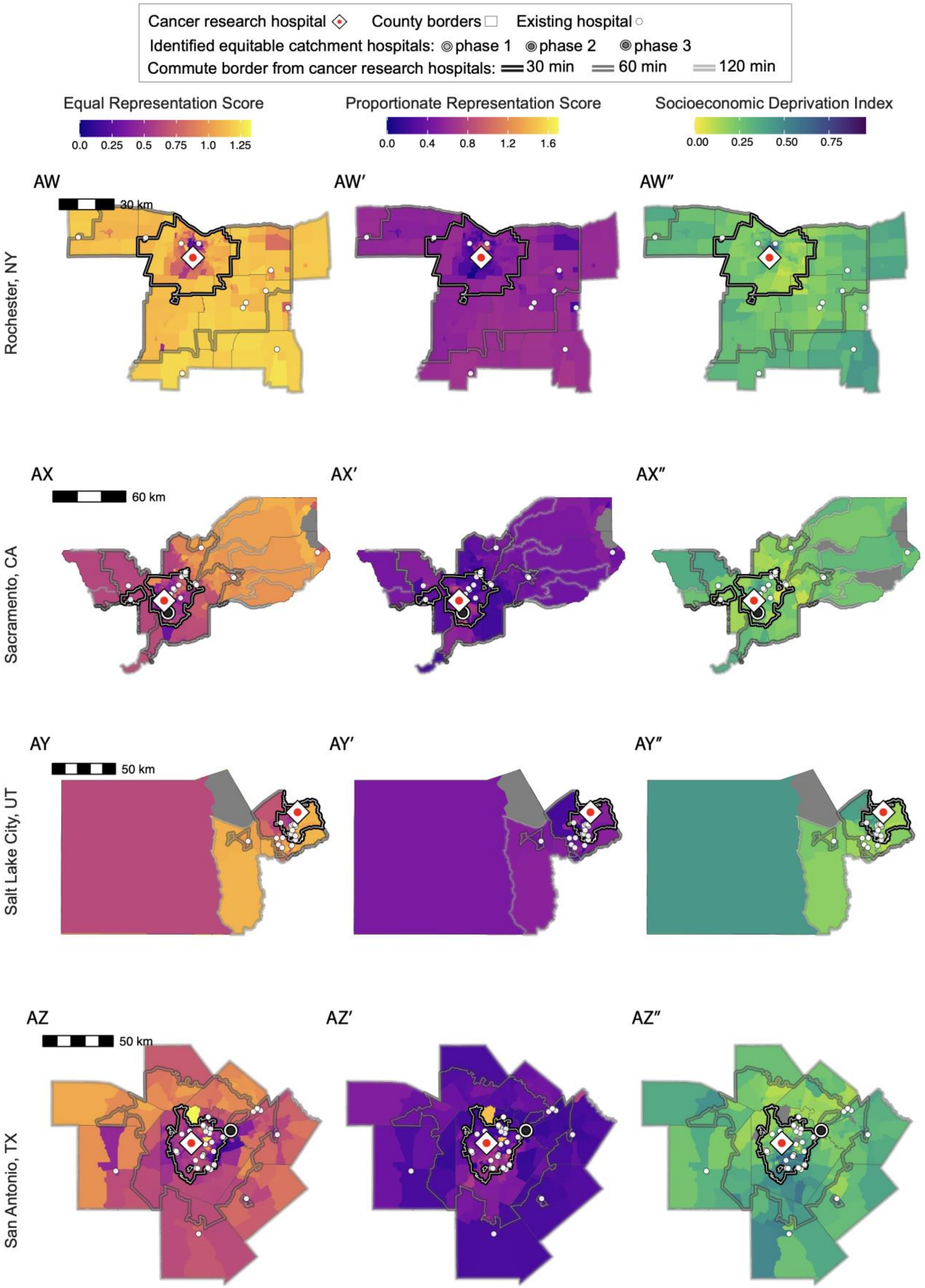

© 2024 Lee H et al. *JAMA Oncology.*

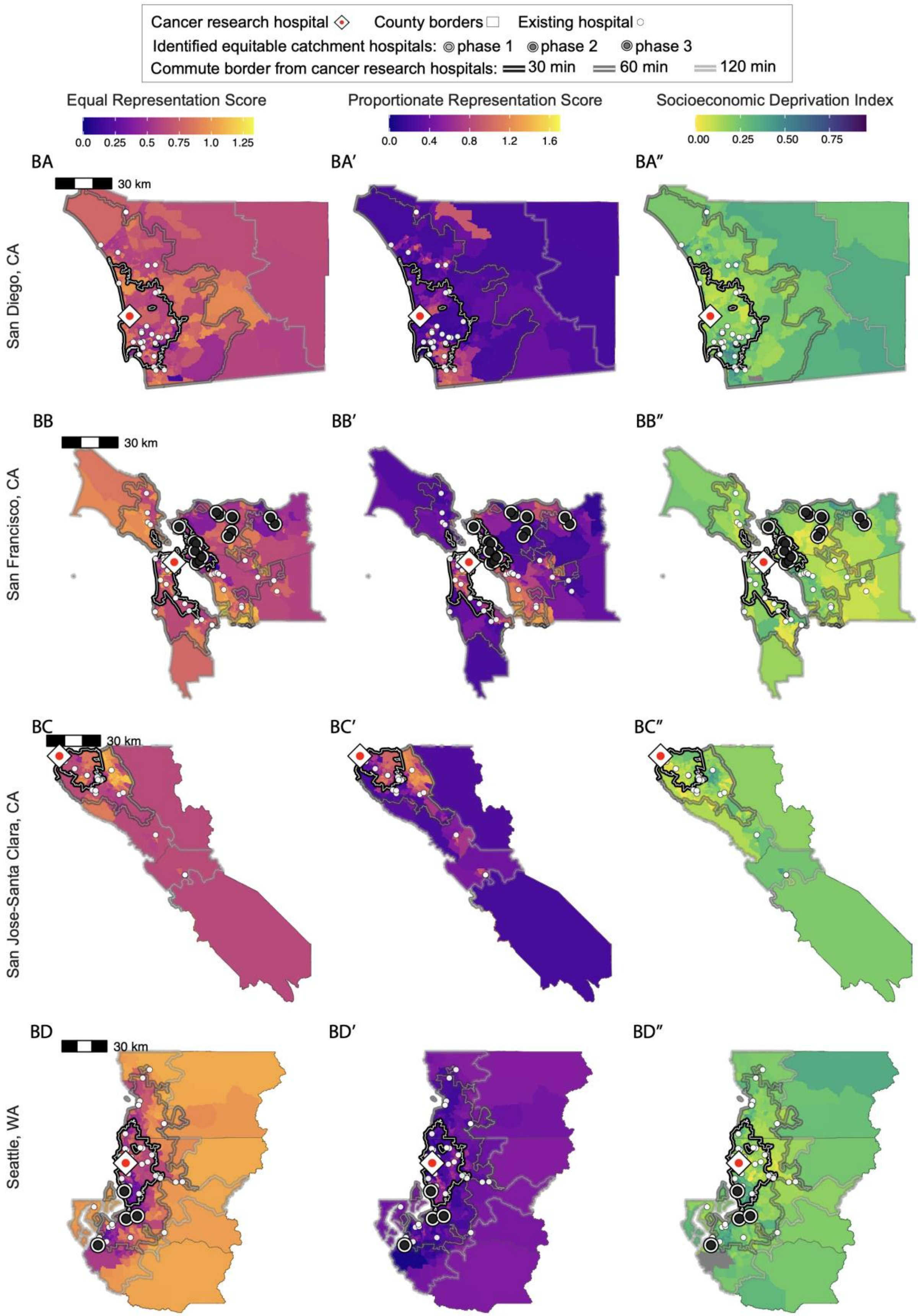

© 2024 Lee H et al. *JAMA Oncology.*

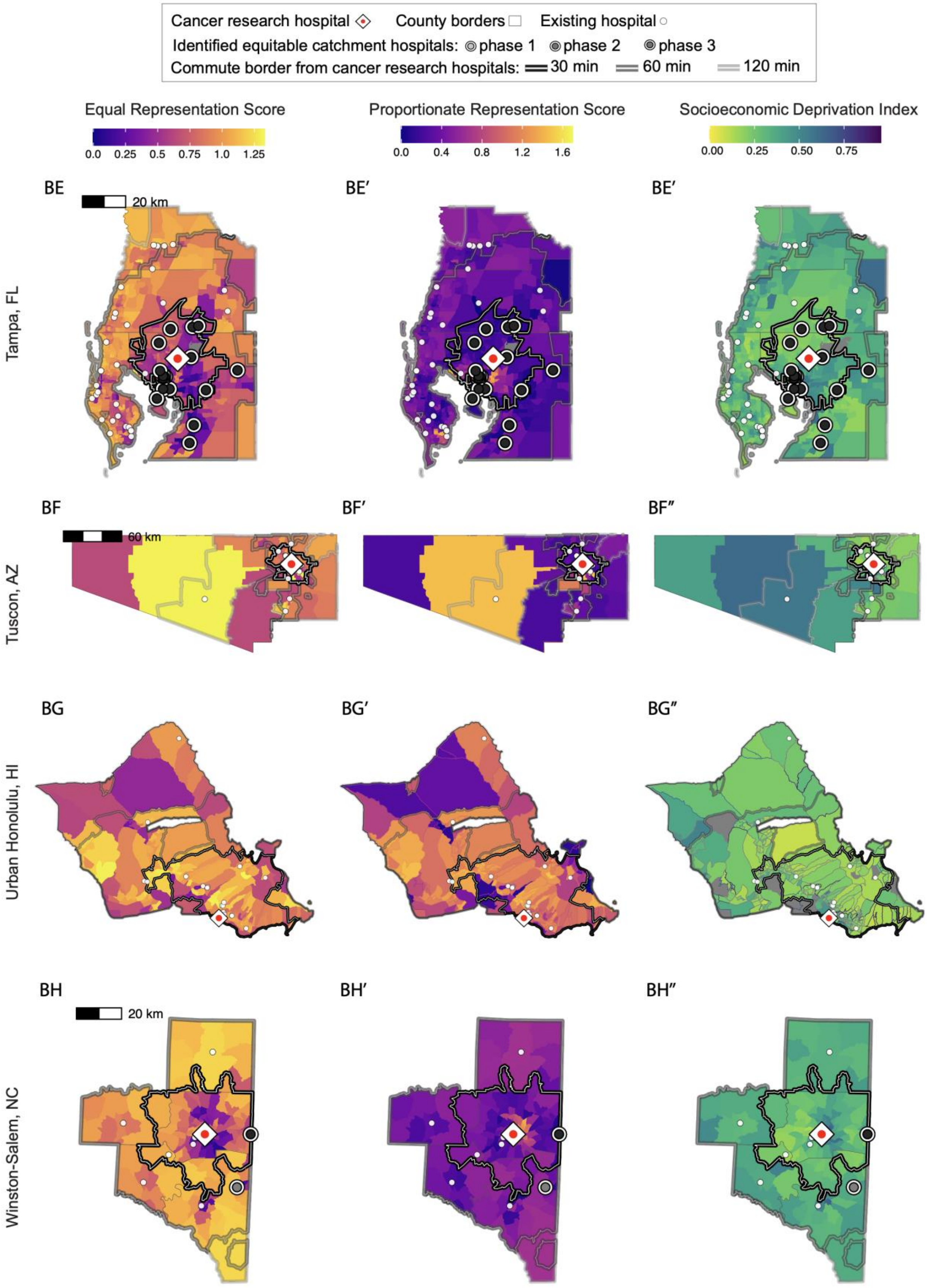

© 2024 Lee H et al. *JAMA Oncology.*

eTable 1. Quantitative summary of U.S. cancer clinical trial sites

			Funding			Phase		
Number of Trial Sites	Number of Trials	Number of Participants	NIH	Industry	Other	1	2	3
78	12,156	2,372,817	3,071 (25.3%)	7,003 (57.6%)	7,313 (60.2%)	5,601 (46.1%)	7,025 (57.8%)	1,403 (11.5%)

The total numbers of cancer clinical trials, targeted numbers of enrolled patients, percentage breakdown of funding sources, and trial phase (1–3) are provided for all trials registered from all 78 U.S.-based clinical cancer research sites between 2012–2022. Multiple assignment of clinical trials to certain subcategories is possible (e.g., a Phase I/II trial with combined industry and NIH funding would be counted in both the 'Industry' and 'NIH' funding categories). Data at the trial center level are displayed in eTable 2.

© 2024 Lee H et al. *JAMA Oncology.*

eTable 2. List and quantitative summary of individual U.S. cancer clinical trial sites in alphabetical order

			Funding			Phase		
Center	Number of Trials	Number of Participants	NIH	Industry	Other	1	2	3
Abramson Cancer Center, Penn/UPenn	984	363,891	288 (29.3%)	591 (60.1%)	466 (47.4%)	379 (38.5%)	490 (49.8%)	282 (28.7%)
Alvin J. Siteman Cancer Center/Washington University School of Medicine in St. Louis	1,203	466,232	405 (33.7%)	790 (65.7%)	445 (37.0%)	406 (33.7%)	627 (52.1%)	365 (30.3%)
Arizona Cancer Center	427	239,140	142 (33.3%)	278 (65.1%)	157 (36.8%)	102 (23.9%)	189 (44.3%)	188 (44.0%)
Case Comprehensive Cancer Center/Case Western Reserve University Hospital	474	212,140	272 (57.4%)	132 (27.8%)	326 (68.8%)	143 (30.2%)	256 (54.0%)	141 (29.7%)
City of Hope Comprehensive Cancer Center/Beckman Research Institute	1,130	353,270	444 (39.3%)	663 (58.7%)	439 (38.8%)	523 (46.3%)	554 (49.0%)	229 (20.3%)
Cleveland Clinic - Main Campus	890	522,104	311 (34.9%)	523 (58.8%)	408 (45.8%)	249 (28.0%)	466 (52.4%)	309 (34.7%)
Dana Farber/Harvard Cancer Center/Brigham and Women's Hospital/Beth Israel Deaconess Medical Center	2,261	561,538	443 (19.6%)	1,640 (72.5%)	1041 (46.0%)	981 (43.4%)	1260 (55.7%)	389 (17.2%)
Department of Medicine, University of Chicago	1,010	401,307	330 (32.7%)	639 (63.3%)	372 (36.8%)	341 (33.8%)	536 (53.1%)	273 (27.0%)

© 2024 Lee H et al. *JAMA Oncology.*

eTable 2. List and quantitative summary of individual U.S. cancer clinical trial sites in alphabetical order

			Funding			Phase		
Center	Number of Trials	Number of Participants	NIH	Industry	Other	1	2	3
Duke University Hospital/Duke Cancer Institute	709	432,003	199 (28.1%)	502 (70.8%)	242 (34.1%)	216 (30.5%)	361 (50.9%)	242 (34.1%)
Fox Chase Cancer Center, Temple University	583	408,728	169 (29.0%)	390 (66.9%)	224 (38.4%)	200 (34.3%)	275 (47.2%)	196 (33.6%)
H. Lee Moffitt Cancer Center & Research Institute - Magnolia Campus	1,008	253,461	195 (19.3%)	772 (76.6%)	364 (36.1%)	426 (42.3%)	550 (54.6%)	206 (20.4%)
Harold C. Simmons Comprehensive Cancer Center/The University of Texas Southwestern Medical Center	697	470,853	208 (29.8%)	430 (61.7%)	310 (44.5%)	186 (26.7%)	326 (46.8%)	294 (42.2%)
Health Sciences Center, University of Louisville	549	342,300	161 (29.3%)	365 (66.5%)	193 (35.2%)	104 (18.9%)	237 (43.2%)	272 (49.5%)
Holden Comprehensive Cancer Center, University of Iowa	474	380,309	221 (46.6%)	257 (54.2%)	226 (47.7%)	106 (22.4%)	224 (47.3%)	202 (42.6%)
Hollings Cancer Center /Medical University of South Carolina	451	439,611	183 (40.6%)	245 (54.3%)	233 (51.7%)	85 (18.8%)	180 (39.9%)	229 (50.8%)
Huntsman Cancer Institute/University of Utah	820	396,142	264 (32.2%)	505 (61.6%)	360 (43.9%)	240 (29.3%)	382 (46.6%)	310 (37.8%)

© 2024 Lee H et al. *JAMA Oncology.*

eTable 2. List and quantitative summary of individual U.S. cancer clinical trial sites in alphabetical order

			Funding			Phase		
Center	Number of Trials	Number of Participants	NIH	Industry	Other	1	2	3
Indiana University School of Medicine	571	388,542	168 (29.4%)	377 (66.0%)	280 (49.0%)	152 (26.6%)	297 (52.0%)	196 (34.3%)
Joan and Sanford I. Weill Department of Medicine, Cornell University	675	404,744	131 (19.4%)	498 (73.8%)	259 (38.4%)	241 (35.7%)	351 (52.0%)	196 (29.0%)
Massachusetts General Hospital	1,400	415,825	249 (17.8%)	1,027 (73.4%)	622 (44.4%)	551 (39.4%)	773 (55.2%)	285 (20.4%)
Mayo Clinic Comprehensive Cancer Center, Rochester, MN	906	523,098	381 (42.1%)	519 (57.3%)	362 (40.0%)	244 (26.9%)	460 (50.8%)	337 (37.2%)
Mays Cancer Center at University of Texas Health San Antonio/The University of Texas Health Science Center at San Antonio	603	412,891	138 (22.9%)	445 (73.8%)	171 (28.4%)	266 (44.1%)	239 (39.6%)	193 (32.0%)
Memorial Sloan Kettering Cancer Center	2,113	713,662	427 (20.2%)	1,379 (65.3%)	1,022 (48.4%)	929 (44.0%)	1,158 (54.8%)	371 (17.6%)
Mount Sinai Health System - Icahn School of Medicine	613	272,820	127 (20.7%)	461 (75.2%)	205 (33.4%)	227 (37.0%)	283 (46.2%)	201 (32.8%)
O'Neal Comprehensive Cancer Center	617	426,279	209 (33.9%)	411 (66.6%)	226 (36.6%)	205 (33.2%)	313 (50.7%)	204 (33.1%)
Ohio State University Wexner Medical Center	815	499,764	300 (36.8%)	498 (61.1%)	345 (42.3%)	223 (27.4%)	404 (49.6%)	301 (36.9%)

© 2024 Lee H et al. *JAMA Oncology.*

eTable 2. List and quantitative summary of individual U.S. cancer clinical trial sites in alphabetical order

			Funding			Phase		
Center	Number of Trials	Number of Participants	NIH	Industry	Other	1	2	3
University of Oklahoma Health Sciences Center	646	358,687	310 (48.0%)	334 (51.7%)	318 (49.2%)	170 (26.3%)	276 (42.7%)	286 (44.3%)
Robert H. Lurie Comprehensive Cancer Center/Department of Medicine, Northwestern University	853	324,939	324 (38.0%)	511 (59.9%)	373 (43.7%)	270 (31.7%)	477 (55.9%)	250 (29.3%)
Roswell Park Comprehensive Cancer Center	733	325,355	262 (35.7%)	431 (58.8%)	356 (48.6%)	278 (37.9%)	367 (50.1%)	222 (30.3%)
Sidney Kimmel Comprehensive Cancer Center/Johns Hopkins University	1,095	328,938	383 (35.0%)	629 (57.4%)	549 (50.1%)	404 (36.9%)	612 (55.9%)	233 (21.3%)
Stanford Cancer Institute/Stanford Department of Medicine	918	460,537	269 (29.3%)	584 (63.6%)	410 (44.7%)	303 (33.0%)	477 (52.0%)	286 (31.2%)
The Barbara Ann Karmanos Cancer Institute	881	513,267	296 (33.6%)	575 (65.3%)	284 (32.2%)	305 (34.6%)	431 (48.9%)	294 (33.4%)
The University of Chicago Comprehensive Cancer Center	926	377,202	323 (34.9%)	587 (63.4%)	326 (35.2%)	310 (33.5%)	490 (52.9%)	255 (27.5%)
The University of Texas MD Anderson Cancer Center/The University of Texas Health Science Center at Houston	3,322	841,418	864 (26.0%)	2,163 (65.1%)	1,530 (46.1%)	1,536 (46.2%)	1,865 (56.1%)	525 (15.8%)

© 2024 Lee H et al. *JAMA Oncology.*

eTable 2. List and quantitative summary of individual U.S. cancer clinical trial sites in alphabetical order

			Funding			Phase		
Center	Number of Trials	Number of Participants	NIH	Industry	Other	1	2	3
The Winship Cancer Institute of Emory University	928	354,046	271 (29.2%)	654 (70.5%)	366 (39.4%)	346 (37.3%)	471 (50.8%)	264 (28.4%)
UC Davis Comprehensive Cancer Center	441	279,908	256 (58.0%)	192 (43.5%)	158 (35.8%)	133 (30.2%)	175 (39.7%)	194 (44.0%)
UC San Diego - Moores Cancer Center	585	329,312	150 (25.6%)	431 (73.7%)	190 (32.5%)	174 (29.7%)	275 (47.0%)	223 (38.1%)
UCLA Health Jonsson Comprehensive Cancer Center	1,251	546,243	248 (19.8%)	970 (77.5%)	334 (26.7%)	460 (36.8%)	600 (48.0%)	382 (30.5%)
UCSF Helen Diller Family Comprehensive Cancer Center	936	364,231	300 (32.1%)	614 (65.6%)	417 (44.6%)	359 (38.4%)	463 (49.5%)	260 (27.8%)
UNC Lineberger Comprehensive Cancer Center	594	403,850	250 (42.1%)	316 (53.2%)	320 (53.9%)	141 (23.7%)	308 (51.9%)	200 (33.7%)
University of Cincinnati/ Cincinnati Children's Hospital Medical Center	613	426,195	235 (38.3%)	346 (56.4%)	290 (47.3%)	190 (31.0%)	256 (41.8%)	255 (41.6%)
University of Colorado Cancer Center	966	568,380	276 (28.6%)	669 (69.3%)	289 (29.9%)	358 (37.1%)	430 (44.5%)	333 (34.5%)
University of Maryland Marlene and Stewart Greenebaum Comprehensive Cancer Center	752	361,703	248 (33.0%)	484 (64.4%)	275 (36.6%)	187 (24.9%)	373 (49.6%)	296 (39.4%)

© 2024 Lee H et al. *JAMA Oncology.*

eTable 2. List and quantitative summary of individual U.S. cancer clinical trial sites in alphabetical order

			Funding			Phase		
Center	Number of Trials	Number of Participants	NIH	Industry	Other	1	2	3
University of Rochester Medical Center	441	426,983	201 (45.6%)	238 (54.0%)	212 (48.1%)	68 (15.4%)	185 (42.0%)	240 (54.4%)
University of Wisconsin Carbone Cancer Center	615	470,271	281 (45.7%)	323 (52.5%)	271 (44.1%)	145 (23.6%)	309 (50.2%)	244 (39.7%)
Hillman Cancer Center/University of Pittsburgh Medical Center	1,027	429,266	357 (34.8%)	651 (63.4%)	367 (35.7%)	371 (36.1%)	509 (49.6%)	307 (29.9%)
USC Norris Comprehensive Cancer Center/University of Southern California	680	416,222	239 (35.1%)	446 (65.6%)	200 (29.4%)	212 (31.2%)	345 (50.7%)	236 (34.7%)
University of Washington School of Medicine - Fred Hutchinson Cancer Center/Seattle Cancer Care Alliance	881	457,399	324 (36.8%)	542 (61.5%)	411 (46.7%)	286 (32.5%)	484 (54.9%)	260 (29.5%)
Vanderbilt-Ingram Cancer Center/Vanderbilt University Medical Center	829	471,160	254 (30.6%)	551 (66.5%)	287 (34.6%)	252 (30.4%)	433 (52.2%)	272 (32.8%)
Wake Forest Baptist Comprehensive Cancer Center	476	405,963	269 (56.5%)	224 (47.1%)	245 (51.5%)	89 (18.7%)	225 (47.3%)	210 (44.1%)
Yale Cancer Center	689	357,846	186 (27.0%)	497 (72.1%)	194 (28.2%)	250 (36.3%)	286 (41.5%)	256 (37.2%)

The total numbers of cancer clinical trials, patients enrolled per protocol, percentage breakdown of funding sources, and trial phase (1–3) is provided for the all 78 U.S. clinical cancer trial sites based on data from registered trials on ClinicalTrials.gov between 2012–2022. Centers are listed in alphabetical order. Multiple allocations of clinical trials are possible (e.g., a Phase I/II trial with combined industry and NIH funding would be counted in both the ‘Industry’ and ‘NIH’ funding categories).

© 2024 Lee H et al. *JAMA Oncology.*

eMethods: Unabridged Data Sources, Calculations and Analysis

Data sources

A list of major U.S. cancer clinical trial centers (N = 78) were collated from two data sources, the nature-index top 100 cancer research healthcare institutions list[1] and non-laboratory based National Cancer Institute Comprehensive Cancer Centers[2] (eFig1). Trial volume for each institution between 2012-2022 was queried on the national trial registry (nct.gov) filtered by age (>18), location (U.S.), trial type (phase 1, 2, 3) and recruitment status (excluded if 'terminated' or 'withdrawn'). Trials with more hospital sites registered than number of patients were excluded. The major U.S. cancer trial centers (N = 78) were found to be listed in 94% of all U.S. cancer trials.

The geographic locations of all hospitals in the U.S. (N=7,623) were taken from The Homeland Infrastructure Foundation-Level Data[3]. U.S. census data were collected from the 2020 decennial survey and the 5-year American Community Surveys (ACS) for the years 2016-2020, 2011-2015 and 2006-2010 using the *tidycensus* R package[4] for the following variables: self-identified race (P1_001N-P1_009N), fraction vacant housing (B25002_003), assisted income (B19058_002), health insurance status (B27010_{017,033,050,066}), median income (B19013_001), high school education (B15003_017-B15003_025), and poverty level (B17001_002) per census tract. The U.S. census bureau provided geographical crosswalk data file (https://www2.census.gov/geo/docs/maps-data/data/rel2020/tract/tab20_tract20_tract 10_natl.txt) was downloaded to correlate historical 2010 census tracts to updated 2020 census tracts. This resulted in re-identification of 96.5% of called 2020 census tracts within historical census data.

Racial Diversity Score

Diversity data were obtained by retrieving self-identified race population counts from the 2020 US decennial census. A diversity score was calculated as the sum of the absolute difference of the catchment population from an equal 1/3rd White, Black/African American, and Asian/Mixed/Other race representation, with values closer to 0 indicating a closer to equal 1/3rd representation. These groupings were selected because they represent the 3 numerically largest non-overlapping U.S. populations and captured the range of diverse representation in the high volume cancer trial sites (Fig 1D).

Catchment population identification

The *osrm* R package (Open StreetMap[5,6]) was used to simulate driving times to the centroids of all U.S. census tracts located within a 200km flying-distance to chosen hospital or cancer research sites. Catchment populations were defined as the combination of all tracts with centroids located within 30-, 60-, or 120-minute one-way driving distance away from chosen hospital or cancer research site locations.

Threshold population sizes for catchment populations

The threshold size was calculated as the mean number of patients recruited for interventional phase 1, 2, or 3 trials between 2012–2022 from the 78 most active cancer clinical trial sites as described in the data sources section (mean 126, 250, 1,321; standard error 3, 13, 48, respectively), multiplied by the prevalence of all cancers in the U.S. population (5.2% in 2020)[7], multiplied by the national average enrollment rate of adult cancer patients into clinical trials (6.3% in 2021)[8]. Calculated in this manner, the threshold size represents a reasonable expectation of the minimum catchment population size required to recruit sufficient numbers of participants, given cancer incidence and enrollment rates for an active trial site.

Software and Packages

All analyses were conducted and visualized in R studio (v2022.02.1+461) and R (v4.1.3) using *Tidyverse*[9] and *ggplot2.* Two-group estimation plots were produced using *dabestr*[10]. Topologically Integrated Geographic

© 2024 Lee H et al. *JAMA Oncology.*

Encoding and Referencing system (TIGER)/Line shapefiles of the legal boundaries of U.S. census tracts and counties were collected using the *Tigris* R package[11].

<u>References</u>

1. Top 100 healthcare institutions in cancer research | Nature Index 2020 Cancer | Supplements | Nature Index. Accessed October 2, 2022. https://www.nature.com/nature-index/supplements/nature-index-2020-cancer/tables/healthcare
2. NCI-Designated Cancer Centers - NCI. Accessed October 2, 2022. https://www.cancer.gov/research/infrastructure/cancer-centers
3. HIFLD Open Data. Accessed October 2, 2022. https://hifld-geoplatform.opendata.arcgis.com/search?collection=Dataset
4. Walker K, Herman M. tidycensus: Load US Census Boundary and Attribute Data as "tidyverse" and 'sf'-Ready Data Frames. *R package version 123*. Published online 2022. Accessed October 2, 2022. https://walker-data.com/tidycensus/)
5. OpenStreetMap. Accessed October 2, 2022. https://www.openstreetmap.org/about
6. Giraud T. osrm: Interface Between R and the OpenStreetMap-Based Routing Service OSRM. *J Open Source Softw*. 2022;7(78):4574. doi:10.21105/JOSS.04574
7. Cancer of Any Site — Cancer Stat Facts. National Cancer Institute, Surveillance, Epidemiology, and End Results Program. Accessed October 23, 2023. https://seer.cancer.gov/statfacts/html/all.html
8. Unger JM, Fleury M. Nationally representative estimates of the participation of cancer patients in clinical research studies according to the commission on cancer. *https://doi.org/101200/JCO20203928_suppl74*. 2021;39(28_suppl):74-74. doi:10.1200/JCO.2020.39.28_SUPPL.74
9. Wickham H, Averick M, Bryan J, et al. Welcome to the Tidyverse. *J Open Source Softw*. 2019;4(43):1686. doi:10.21105/JOSS.01686
10. Ho J, Tumkaya T, Aryal S, Choi H, Claridge-Chang A. Moving beyond P values: data analysis with estimation graphics. *Nature Methods 2019 16:7*. 2019;16(7):565-566. doi:10.1038/s41592-019-0470-3
11. Walker K. Tigris: An r package to access and work with geographic data from the us census bureau. *R Journal*. 2016;8(2):231-242. doi:10.32614/RJ-2016-043

© 2024 Lee H et al. *JAMA Oncology*.
