# Supplementary material for: Analysis and Optimization of Equitable US Cancer Clinical Trial Center Access by Travel Time
Source: JAMA Oncol. 2024 Mar 21;10(5):652–7. doi: 10.1001/jamaoncol.2023.7314 (PMC10958387; doi:10.1001/jamaoncol.2023.7314)
Supplement: Supplement 2. — Data Sharing Statement [file jamaoncol-e237314-s002.pdf]

Data Sharing Statement

Lee. Analysis and Optimization of Equitable US Cancer Clinical Trial Center Access by Travel Time. *JAMA Oncol*. Published March 21, 2024. doi:10.1001/jamaoncol.2023.7314

Data
Data available: No

Additional Information
Explanation for why data not available: The data used for this study are available to the public. The code and analysis will be published for open access on github.
